# Gene Therapy of Beta Hemoglobinopathies

**DOI:** 10.3390/biomedicines13123093

**Published:** 2025-12-15

**Authors:** Ugo Testa, Elvira Pelosi, Germana Castelli

**Affiliations:** Department of Oncology, Istituto Superiore di Sanità, Viale Regina Elena 299, 00161 Rome, Italy; elvirapelosi56@gmail.com (E.P.); germana.castelli@iss.it (G.C.)

**Keywords:** hemoglobin, gene therapy, sickle cell disease, beta thalassemia, stem cell transplantation, gene editing

## Abstract

**Background/Objectives**: Sickle cell disease (SCD) and β-thalassemia are autosomal recessive disorders of erythroid cells due to gene mutations occurring at the level of the β-globin gene. The severe forms of these hemoglobinopathies observed in individuals homozygous for these defective genes need intensive treatments, are associated with a poor quality of life, and allogeneic hematopoietic stem cell represents the only curative treatment option that can be offered to a limited proportion of patients. **Methods**: This work is a narrative review supported by a systematic literature search and analysis. **Results**: To bypass this limitation, autologous hematopoietic stem cell transplantation has been developed in these patients, in which patients’ HSCs are harvested and genetically modified ex vivo, then transplanted back into patients after conditioning for stem cell transplantation. There are two different approaches for gene therapy of hemoglobinopathies, one based on gene addition or gene silencing using lentiviruses as vectors and the other based on gene editing strategies using CRISPR-Caspase 9 technology or base editing. Several gene therapy products have been successfully evaluated in these patients, achieving transfusion independence and correction of hematological abnormalities durable over time. **Conclusions**: Several gene therapy products have been approved for the treatment of SCD and β-thalassemic patients and offer potentially curative treatment for these patients.

## 1. Introduction

Hemoglobin is a key functional protein of red blood cells (RBCs), constituted by a tetramer α_2_β_2_ bound to a heme group; this protein plays a unique and fundamental role as an O_2_ transporter in the body. The level of globin synthesis markedly increases during erythroid differentiation/maturation and is finely controlled through the modulation of the transcriptional activity of α- and β-globin genes. α- and β-globin genes are organized in gene clusters located at the level of chromosomes 16 and 11, respectively.

**Figure 1:**
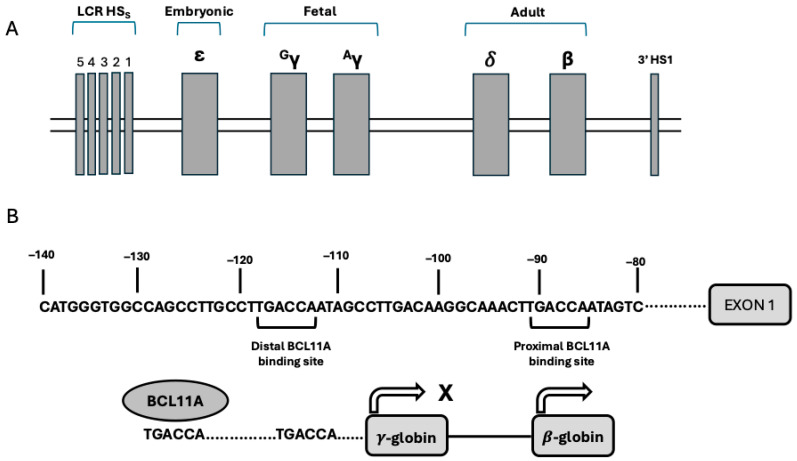
(**A**) Genomic organization of the β-globin gene cluster on chromosome 11. The genes are physically arranged in order of their expression during development, including ε-globin (embryonic), ^G^γ- and ^A^γ-globins (fetal), and δ and β-globins (adult). LCR corresponds to the locus control region (LCR), with its five hypersensitivity sites. HS1 denotes the hypersensitivity site 1. (**B**) Control of γ-globin gene expression mediated by BCL11A. **Top** panel: nucleotide sequence of the gene promoter of *HBG1* and *HBG2* genes: in these promoters, two BCL11A TGACCA binding sites are present (a distal −118 to −113 and a proximal −91 to −86). **Bottom** panel: the distal site actively binds BCL11A in adult erythroid cells and represses γ-globin gene expression.

The HBA (α-globin) locus contains an embryonic gene (ξ-globin) and two adult genes, HBA1 (α_1_-globin) and HBA2 (α_2_-globin); the expression of these genes is controlled by distal enhancers that are active in erythroid cells at various stages of development [1]. The HBB locus (β-globin gene cluster) is composed of 5 β-like globin genes [ε (HBE), γ_2_ (HBG2), γ_1_ (HBG1), δ (HBGD), and β (HBB)]. The physical arrangement of these genes corresponds to their developmental expression: during the embryonic life, the yolk sac primarily synthesizes embryonic hemoglobin (HBE, α_2_ε_2_); around the second month of development, transcription of β-like genes shifts to the γ-globin genes (HBG1, α_2_γ1_2_ and HBG2, α_2_γ2_2_) and the main site of erythroid cell production becomes the liver; at later stages of development, γ-globin chain synthesis is progressively replaced by β-globin chain synthesis (HbF to HbA hemoglobin switching) and this process is fully completed only after birth at 9–12 months of age [1] (Figure 1). The activation of expression of various β-globin-like genes is controlled by the upstream locus control region (LCR), an enhancer element active at all stages of erythroid development. LCR controls globin genes through physical interaction with the promoters of individual globin genes by looping out the intervening DNA; this looping is mediated by DNA-binding proteins and transcription factors that bridge the LCR and promoters, bringing them in contact, despite their consistent physical distance. These interactions between LCR and specific promoters play a key role in activating gene transcription at a given developmental stage [1]. Thus, the LCR loops to the γ-globin genes in fetal erythroid and not to the β-globin gene in adult erythroid cells, resulting in their reciprocal expression. Enforced looping that generates contact between the LCR and the γ-globin gene promoters determines high levels of γ-globin expression and reduced β-globin expression [1] (Figure 1).

Silencing of the globin gene is controlled by the promoter regions of developmentally expressed globin genes. An important example of these silencing mechanisms is given by the repression of HBG gene expression in adult erythroid cells mediated through the recruitment of repressor proteins to the HBG promoters with BCL11A binding 115 bp upstream of the transcription start site [1] (Figure 1).

BCL11A is a zinc-finger protein predominantly expressed in the brain and in erythroid cells. The BCL11A expression is transcriptionally controlled during development: in fetal erythroid cells, BCL11A expression is inhibited by the repressor HIC2, whose expression is high in fetal cells but low in adult erythroid cells [2]. The expression of BCL11A in erythroid cells is controlled by an erythroid-specific enhancer located at position +58 in intron 2 of the BCL11A gene [3]. The +58 BCL11A intronic enhancer contains a binding site for GATA1, which is required to sustain erythroid-specific expression of BCL11A [3].

In addition to BCL11A, another repressor, the LRF/ZBTB7A transcription factor, inhibits γ-globin gene transcription in adult erythroid cells [4]. This factor binds to a specific binding element present in the γ-globin promoter at −197 [4]. The mutation −198T > C is responsible for a form of hereditary persistence of fetal hemoglobin (HPFH), generating a binding site for the HbF activator KLF1. BCL11A and ZBTB7A independently repress expression of HbF.

Mutations in the β-globin gene cause two autosomal recessive diseases, sickle cell disease (SCD) and β-thalassemia. In SCD, a point mutation of the β-globin gene which determines the substitution of glutamic acid at position 6 with a valine residue results in the production of an abnormal hemoglobin (HbS) that in its deoxygenated form tends to polymerization; this polymerization of HbS alters the architecture and flexibility of the sickle RBCs with increased hemolysis and tendency of veno-occlusive events with consequent tissue damage [5]. β-thalassemias are characterized by reduced/absent β-globin chain synthesis due to more than 400 different mutations at the level of the β-globin gene cluster, including point mutations, minor deletions or insertions, or gross deletions in either the β-globin gene or in its flanking, noncoding regions. HbA is a tetramer α_2_β_2_ composed of 2 α and two β chains and a heme prosthetic group that binds an iron atom (Fe_2_), which is essential for its function; the tetramer is a functional, physically stable structure, while free globin chains are unstable and have the tendency to precipitate. The reduced/absent β-globin chain synthesis results in a markedly decreased/absent synthesis of HbA, with an excess of free α-globin chains that are unstable, precipitate, and induce oxidative damage with alterations of the membrane of RBCs and consequent premature death (dyserythropoiesis) [6]. The only possible curative effect for both SCD and β-thalassemia patients is represented by allogeneic HSCT. Allo-HSCT allows for a one-time cure of SCD or β-thalassemia; however, only a minority (about 25%) of these patients can find a suitable donor due to immunoincompatibility and limited accessibility.

Allo-HSCT in SCD patients offers a high rate of survival using HLA-identical sibling donors; however, best outcomes were observed only among children under 5 years (with a 5-year overall survival (OS) and event-free survival (EFS) of 99% and 96%, respectively), while patients over 15 years had OS and EFS of 88% and 84%, respectively [7]. To increase the number of SCD patients suitable for HSCT, alternative HSCT donors have been used, including related HLA-mismatched or haploidentical, unrelated HLA-identical, and unrelated HLA-mismatched individuals. The improvement in the conditioning regimens and the introduction of efficient pharmacologic strategies of immunosuppression and prophylaxis of graft-versus-host disease (GVHD) have shown promising results in recent studies of allo-HSCT in SCD patients using haploidentical donors [8,9]. However, allo-HSCT for SCD patients using not fully HLA-compatible donors remains challenging and available only in specialized medical centers; graft rejection, acute/chronic GVHD, transplant-related toxicity, and reduced benefit in adults with organ damage represent key limitations/complications of allo-HSCT in these patients.

In β-thalassemic patients, allo-HSCT using HLA-matched sibling donors determines, after engraftment, the development of normal hematopoiesis, progressively replacing β-thalassemic erythropoiesis, reducing hemolysis, improving erythroid maturation, increasing hemoglobin levels, and eliminating the need for blood transfusions. Analysis of the international registry of β-thalassemia patients showed an OS for patients who underwent allo-HSC under the age of 14 years of 94–96%, while for adult patients with more advanced disease and hepatic damage related to iron overload, the OS does not exceed 80% [10]. The improvement of allo-HSCT procedures has allowed the expansion of allo-HSCT of β-thalassemic patients to HLA-mismatched donors. Advances in conditioning regimens, pharmacologic immunosuppression, and T-cell depletion strategies have fostered high rates of OS and thalassemia-free survival in haplo-HSCT [11].

To bypass these limitations, autologous HSC gene therapy was developed, in which patients’ HSCs are harvested and genetically modified ex vivo, then transplanted back into the patient after conditioning with busulfan or other cytotoxic agents. Basically, there are two distinct approaches for gene therapy of hemoglobinopathies: one approach is based on gene addition strategies based on lentiviral vectors to add functional copies of the gene encoding β-globin in defective HSCs or to silence faulty genes such as BCL11A to induce HbF synthesis to replace or to counteract the defective β-globin; the second approach is based on gene editing strategies involving the use of CRISPR-Cas9, transcription activator-like effector protein nuclease and zinc finger nuclease techniques either for directly repair the underlying genetic cause of disease or to induce HbF production by gene disruption [12,13].

## 2. Gene Therapy Strategies

Basically, there are two main approaches for gene therapy for the treatment of hereditary hemoglobinopathies. One approach is based on gene insertion, a procedure in which a therapeutic globin gene is introduced into hematopoietic stem cells. In this approach, patients continue to express the pathologic β-thalassemic or βS globin gene, and the therapeutic gene inserted determines the synthesis of a globin gene competing with the pathologic β-gobin gene, inducing the generation of a scenario similar to that observed in patients with β-thalassemic trait or with the sickle cell trait. Alternatively, the gene inserted in HSCs encodes the synthesis of a gene, such as an RNA interfering with the BCL11A gene, thus reactivating HbF synthesis that counteracts HbS or replaces defective HbA synthesis in β-thalassemic patients. The second approach is based on gene editing through genetic procedures, causing disruption of an HbF repressor or correction of the disease-causing mutation.

In the gene therapy approaches by gene insertion, it is of fundamental importance to evaluate the minimal percentage of the transgene that needs to be expressed in vivo in comparison with the pathologic β-globin; furthermore, an additional important determinant is also the cellular distribution of the therapeutic transgene at the level of RBCs. Considering clinical experience with hemoglobinopathy patients treated with hematopoietic stem cell transplantation, it was evaluated that a minimal expression of 20% donor myeloid chimerism is required to reverse the sickle phenotype; in β-thalassemic patients, a lower level around 15% seems to be necessary to correct ineffective erythropoiesis and reverse the anemic condition [14].

It is important to note that the same principle can be applied to gene therapy studies aiming to reactivate HbF synthesis, where a minimum level of HbF synthesis is required to efficiently correct hematopoietic defects observed in SCD or in β-thalassemic patients.

All the procedures of treatment of SCD and β-thalassemic patients with ex vivo genetically manipulated HSCs/HPCs are complex (Table 1) [10]. The initial steps, preceding the transplantation of engineered HSCs/HPCs, including initial evaluation with patient selection and preparation, mobilization of HSCs/HPCs, isolation of HSCs/HPCs, and ex vivo manufacturing, require considerable scientific expertise and a GMP laboratory authorized for the manipulation and culture of human HSCs for clinical use. Importantly, during the initial evaluation, patients are assessed for the availability of a full sibling who would be HLA-matched; in this eventuality, allo-HSCT is evaluated as a valuable therapeutic option [10]. The subsequent clinical steps of administration of conditioning chemotherapy, transfusion of engineered autologous HSCs/HPCs, and monitoring of patients post-transplantation require at least 4–6 weeks of hospitalization in a specialized clinical unit [15].

HSC mobilization and collection is a fundamental step in the process of gene therapy and must provide an adequate number of HSCs/HPCs to be ex vivo genetically manipulated. In β-thalassemic patients, the combination of G-CSF (a growth factor that stimulates stem cell production) plus Plerixafor (a CXCR4 inhibitor that promotes the mobilization of stem cells from BM to PB) provides high yields of CD34^+^ cells with a primitive signature, suitable for gene therapy studies [16]. In SCD patients, HSC mobilization is challenging since several SCD-related factors impair HSC mobilization and collection, including the presence of damaged bone marrow, cytotoxic effects exerted by prior therapy with hydroxyurea, and, mostly, the inability to use G-CSF, which can trigger severe vaso-occlusive events [17]. Peripheral blood mobilization using plerixafor followed by apheresis collection represents a safe and effective procedure to obtain adequate numbers of HSCs in most SCD patients [17].

Autologous gene-modified cell therapies to treat β-hemoglobinopathies require a disease-specific conditioning, derived from the experience in myeloablative and nonmyeloablative HSCT for these disorders. In most of the studies of gene therapy of β-hemoglobinopathies, either based on gene addition or gene editing, including the studies on gene therapy products approved for clinical use, myeloablative regimens have been adopted to allow gene-modified cells to engraft and to provide a durable and sufficient therapeutic effect. In these studies, the preferred myeloablative regimen consisted of Busulfan at a dosage comprised between 70 and 90 mg*h/L [18]. Therapeutic drug monitoring is required to minimize toxicities induced by the myeloablative regimen. Several gene therapy studies based on the use of lentiviral vectors have used reduced-intensity conditioning using either sub-myeloablative doses of Busulfan (40–60 mg*h/L) or melphalan; in these studies, positive evidence of the therapeutic effects was usually observed, but not at a curative level [18].

This section analyzes the various clinical studies carried out in patients with hemoglobinopathies and based on a gene addition strategy using lentiviral vectors.

## 3. Gene Therapy of Beta-Hemoglobinopathies by Gene Addition Using Lentiviral Vectors

### 3.1. Different Lentiviral Vectors Used in Clinical Trials for Hemoglobinopathies

Gene therapy approaches of gene addition were based on the use of lentiviral vectors. These vectors offer the advantage of delivering high levels of transgene in populations of quiescent cells, such as hematopoietic stem cells, and of conferring long-term, stable expression of the transduced transgene.

The construction of lentiviral vectors is based on some elements required to ensure a consistent level of safety and a high level of expression in erythroid cells (Table 2). Concerning the safety, to reduce the risk of inappropriate oncogene activation, current lentiviral vectors utilize self-inactivating mechanisms of viral promoter present in the long-terminal repeats (LTR), acting upon vector integration; these inactivation mechanisms limit the risk of inappropriate activation of nearby genes. Particularly, lentiviruses are produced as replication-defective viral particles in which the RNA genome contains a self-inactivating (SIN) 3′LTR, which upon reverse transcription into a linear, double-stranded DNA gene gives rise to a U3-deleted enhancer and promoter-less 5′ LTR [19].

The induction of high expression in erythroid cells requires the presence of internal lineage-specific promoters that drive the hyperexpression of the integrated vectors in erythroid cells; these enhancers are present in the β-LCR and in the intron-2 and 3′ untranslated region of the β-globin gene [20]. The key elements in the β-LCR are represented by five hypersensitive sites, named HS1 to 5. For the construction of a genomically stable vector suitable for globin gene therapy, the presence of DNA sequences encompassing HS2, HS3, and HS4 of the LCR, together with β-globin gene proximal regulatory elements, provides therapeutic levels of β-globin expression [21]. The presence of intron 2 of the β-globin gene in the retroviral vector is fundamental because this region contains an essential enhancer element [22].

Interestingly, through the study of a library of short sequences derived from developmentally active elements in erythropoiesis, a clinically relevant enhancer was identified, replacing the canonical LCR and ensuring therapeutic levels of β-globin transgene expression in a β-thalassemia lentiviral vector [23].

A fundamental element of the lentiviral vector is represented by the transgene. Four different vectors have been used in clinical trials for SCD and β-thalassemia. The choice of the transgene is related to its function and its capacity to replace the pathologic β-globin gene. Concerning gene therapy, there are multiple possible transgenes related to their capacity to efficiently counteract the sickling effect of β^S^; in this context, γ-globin chains possess a strong anti-sickling activity related to their higher oxygen affinity and to structural properties related to glutamine at position 87 of the γ-globin chains. The analysis of the properties of γ-chains in their interaction with β^S^ and with α chains allowed the development of a β-chain mutant incorporating three aminoacidic changes T87Q, E22A, and G16D, allowing the generation of mutated β-chains with anti-sickling properties, similar to those of γ-chains and with an affinity for α chains higher than that of WT β-chains; these three mutations incorporated into a β-chain allowed the generation of β^AS3^ globin, exhibiting enhanced anti-sickling capacities [24,25]. The introduction of a G16D mutation in the γ-globin allowed the generation of a γ-globin chain with enhanced anti-sickling activity [26].

The four different vectors used in gene therapy trials were: a vector expressing a WT β-globin gene (Vebeglogene Autotemcel); a vector expressing a modified β-globin, β^T87Q^ (Levotibiglogene or Betibeglogene); a vector expressing β^AS3^; and a vector expressing a modified γ-globin, γ^G16D^.

Some lentiviral vectors used for gene therapy of hemoglobinopathies contain insulator elements, corresponding to DNA sequences that control the activity of enhancer elements present in these vectors. Thus, Cabriolu and coworkers showed that HS1 and HS2 LCR elements present in the lentiviral vectors can be activated in early hematopoietic cells, including HSCs; this activity may result in inappropriate transcriptional activation of nearby genes [27]. The introduction of an insulator element, such as A1 insulator, reduced non-erythroid expression, while expression in erythroid cells was unaffected; importantly, incorporation of the A1 insulator in a therapeutic globin vector was not detrimental to vector titer and hemoglobin production in globin vector-treated thalassemic mice [27].

The most relevant clinical trials of gene therapy involving the use of lentiviral vectors are reported in Table 3.

### 3.2. Gene Therapy of SCD Using Lovo-Cel

Lovotibeglogene (Lovo-Cel, BB111; Lenti-Globin for SCD) gene therapy is administered by autologous stem cell transplantation with cells transduced with the BB05 lentiviral vector encoding the modified β-globin gene (β^T87Q^), resulting in the production of HbA^T87Q^ protein with anti-sickling properties. The HbA^T87Q^ protein was specifically designed to sterically inhibit HbS polymerization, and preclinical studies have shown the capacity of Lovo-Cel-mediated endogenous expression of β^T87Q^ to inhibit HbS polymerization [24,28].

Interestingly, studies in an erythroid cell line expressing β^S^ showed that expression of β^T87Q^ in these cells induced a significant reduction of β^S^ synthesis [21]. This observation suggests that β^T87Q^ exerts an anti-sickling effect through two different mechanisms, o related to a direct competition of β^T87Q^ with β^S^ and the other related to an inhibitory effect of β^T87Q^ on β^S^ synthesis through an undefined mechanism [29].

The Lenti-Globin was evaluated in patients with SCD in the context of the clinical trial HB-205. Initially, one SCD patient was treated with Lenti-Globin BB305, resulting in persistent expression of the transgene in vivo, with a level of β^T87Q^ corresponding to about 50% of total hemoglobin, with no recurrence of sickle crises [30]. An initial report of the HGB-205 trial included 3 SCD patients: 2/3 patients displayed a long-term clinical response, and 1/3 displayed a reduced transfusional need [31]. After the initial proof of the efficacy and safety of Lenti-Globin-based gene therapy of SCD patients, the phase I/II HGB-206 trial was started using Lenti-Globin BB305 in 50 adults and adolescent patients with severe SCD. In group A, seven patients were treated and achieved only partial therapeutic effect due to inappropriately low levels of HbA^T87Q^ (0.46 g/dL) due to low copy number per cell observed 6 months after gene therapy [32]. To bypass these limitations, the procedure of gene transfer was improved, and Plerixafor mobilization of HPC progenitors was used in patients to improve the recovery of CD34^+^ cells. Using this strategy, higher levels of VCN and of HbA^T87Q^ were obtained in group B of two patients [32]. Using these manufacturing modifications and optimized HPC mobilization, in the phase C, 36 SCD patients were treated with a single autologous infusion of CD34^+^ cells transduced with Lenti-Globin leading to an increase in total hemoglobin levels from 8.5 g per deciliter at baseline to 11g or more from 6 months through 36 months after infusion; HbA^T87Q^ mean levels were 5.2 g/dL and contributed at least 40% of total hemoglobin, with a distribution across over a mean of 85 ± 8% of RBC; VCN was 1.5 copy/cell [33]. All evaluable patients had resolution of severe vaso-occlusive events and reduction in hemolysis [33]. It is important to note that in the HGB-206 trial, the enrollment criteria included only SCD patients who had at least four vaso-occlusive events in the 24 months before enrollment [33]. In 2020, the phase III clinical study HGB-210, involving the enrollment of pediatric patients aged 12–18 years, was initiated. Recent reports provided updated analyses of the clinical data observed in 36 SCD patients enrolled in the context of the HGB-206 study and 19 SCD patients enrolled in the context of the HGB-210 study [34,35]. The results of this combined analysis showed that HbA^T87Q^ levels remained stable during follow-up and were similar in adult and pediatric patients, with a mean percentage of HbA^T87Q^ of 49% (range 26–63%). The large majority of patients had complete resolution of the vascular occlusion episodes, including severe occlusion episodes, in the two years following gene therapy [34,35]. The pharmacodynamic parameters, such as transduction efficiency, the total Hb level, and the HbA^T87Q^ levels, are sensitive and early predictors of the clinical response of SCD patients to Lovo-Cel-based gene therapy [36].

Several studies have evaluated the socioeconomic impact of Lovo-Cell gene therapy in SCD patients. An analysis of cost-effectiveness suggested remarkable improvements in survival, quality of life, and other outcomes for Lovo-Cel compared to common care [29]. The costs associated with Lovo-Cel were, in part, offset by predicted reductions in other health systems and societal costs [37].

Importantly, in December 2023, the FDA approved the use of Lovo-Cel for the treatment of SCD patients who have a history of vaso-occlusive crises.

An ongoing follow-up study (LTF-307) will provide data on the long-term safety and efficacy of Lovo-Cel therapy in SCD patients.

### 3.3. Gene Therapy of SCD Using Lenti/G-βAS3-FB Vector

In 2003, an anti-sickling β-globin variant was developed by Townes’ laboratory. β^AS3^ involves three β-chain substitutions, including the T/Q change present in Lenti-Globin and the substitutions of glutamic acid in codon 22 with alanine (E22A) and of glycine at codon 16 with aspartic acid (G16D). A lentivirus encoding the β^AS3^ and containing also the FB insulator (Lenti/G-βAS3-FB) was shown to be active in a preclinical SCD mouse model with correction of the hematological parameters and was used for clinical studies [38]. A recent study reported the clinical results obtained in the first five treated patients, reporting a significant improvement in only two of these five SCD patients [39]. Given these non-optimal results, the study was closed to future enrollment.

A second study confirmed the results observed in this study. Thus, Sobrino and coworkers in a phase I/II clinical study (NCT 03964472) evaluated the safety and the efficacy of gene therapy of SCD by transplantation of autologous HSCs/HPCs transduced ex vivo with the DREPANOGLOBE lentivirus vector expressing the β^AS3^ globin gene [40]. The study of the first four SCD patients showed variable results from patient to patient, with only 50% of patients achieving correction of the clinical phenotype with the absence of severe vaso-occlusive events [40]. Single-cell transcriptomic studies showed that reduced engraftment of DREPANIGLOBE-modified CD34^+^ cells was associated with an inflammatory signature [40].

### 3.4. Gene Therapy of SCD Using Lentiviral Vectors Encoding HbF^G16D^

A recent study reported the clinical results observed in SCD patients treated with gene addition therapy based on autologous stem cells transduced with a vector expressing a modified γ-globin gene [41]. This gene therapy was based on the use of a γ-globin lentiviral vector, GbG, encoding human ^A^γ globin gene exons and β-globin noncoding (untranslated regions, introns, and promoter) to ensure high expression of the γ-globin gene in adult erythroid cells [41]. This vector was modified, introducing a change at the 16th codon in γ-globin exon 1 to change glycine (G) to aspartic acid (D), generating a vector expressing γ^G16D^ globin [42]. Experimental studies in SCD mice supported a more potent anti-sickling activity of HbF^G16D^ compared to HbA^T87Q^ [34]. Autologous CD34^+^ cells from 7 SCD patients with severe disease were transduced, and at least 4x10^6^ transduced CD34^+^ cells were reinfused to the patients [42]. Before infusion of autologous stem cells, the patients were submitted to a reduced-intensity conditioning regimen using a reduced-intensity dose of melphalan [42]. One year after infusion, an average vector copy number of >0.01 copies per cell was observed. All seven treated patients displayed sustained HbF^G16D^ expression and >80% reduction in severe vaso-occlusive events [42]. The use of reduced-intensity conditioning instead of myeloablative busulfan decreased the duration of thrombocytopenia and neutropenia and length of hospital stay [42]. The most common adverse events were grade 4 thrombocytopenia and grade 4 neutropenia [34]. The results of this trial need to be confirmed in a larger cohort of patients.

### 3.5. Gene Therapy of β-Thalassemia Using Lovo-Cel

Several studies have evaluated the safety and efficacy of Lenti-Globin BB305 in patients with transfusion-dependent β-thalassemia. The HGB-204 and HGB-205 clinical trials evaluated the safety and efficacy of Lenti-Globin 3305 in 22 patients with transfusion-dependent β-thalassemia: autologous CD34^+^ cells were transduced ex vivo with Lenti-Globin BB305 vector and reinfused into patients after myeloablative busulfan conditioning [43]. Efficacy evaluation involved levels of total Hb, of HbA^T87Q^, transfusion requirements, and average vector copy number [43]. All but one of the thirteen β-thal patients with non-β° genotype stopped receiving red cell transfusions; in nine patients with β°/β° genotype or two copies of IVS1-110 mutation, the median transfusion request was decreased by 73%, and transfusions were discontinued in three patients [43]. Adverse events were those typically associated with autologous SCT [43].

Given the good outcomes of this study, two phase III trials, HGB-207 (Northstar-2) and HGB-212 (Northstar-3), were carried out to more carefully evaluate the safety and efficacy of Lenti-Globin in transfusion-dependent β-thalassemic patients [36]. In the HGB-207 study, β-thal patients with non-β°/β° genotype, and in the HGB-212 study, β-thal patients with β°/β°, and β°/β^+IVS-I110^ genotypes, were enrolled. In this study, a hypertransfusion regimen for at least two months prior HSC/HPC mobilization to maintain Hb levels > 11g/dL was adopted; furthermore, an optimized manufacturing process with improved transduction efficiencies was developed, achieving a mean copy vector number after 24 months of 1.99 copies/cell, compared to a VCN of 0.7 in the HGB-204 and 1.3 in the HGB-205 study [37]. In the HGB-207 study, 20 out of 22 patients reached a condition of transfusion independence with a median HbA^T87Q^ level of 8.7 g/dL (ranging from 5.2 to 10.6 g/dL) at 12 months post-infusion and an average total Hb level of 11.7 g/dL [44]. The HGB-212 study enrolled 18 β-thal patients (twelve with β°/β° genotype, three with β°/β^+IVS-I110^ genotype, and three with β^+IVS-I110^/β^+IVS-I110^ genotype); 89% of these patients reached and maintained a condition of transfusion independence [45]. The two patients who did not achieve transfusion independence had genotypes β°/β° and β^IVS−110^/β^IVS−110^ and had significantly lower VCN (0.199 and 0.320, respectively) compared to those who achieved transfusion independence [45]. Since these two patients received a cell product displaying an adequate vector copy number, the low VCN observed 24 months after infusion suggests a reduced in vivo engraftment of transduced cells. There were no serious adverse events directly related to gene therapy and no deaths [45].

Gibson et al. reported post-approval, real-world experience in nine β-thal patients with Beta-Cel gene therapy [46]. All patients reached transfusion-independence. Patients experienced prolonged platelet engraftment time and high platelet transfusion requirements, which were associated with severe bleeding in patients with veno-occlusive disease or HLA Class I alloimmunization [46]. This observation suggests the need for additional studies aiming to mitigate bleeding complications in these patients. In a real-world setting in Germany, Betabeglogene gene therapy resulted in being safe and effective in eight transfusion-dependent non-β°/β° thalassemic patients [47]. All these patients reached and maintained transfusion independence. The safety profile was acceptable, and most of the patients displayed pituitary-endocrine dysfunction; one patient developed depression and anxiety syndrome; one patient developed fatigue [47].

In conclusion, Lovo-Cel represents an effective gene therapy for transfusion-dependent β-thalassemia, achieving high rates of transfusion-independence and a marked increase in hemoglobin levels. The safety profile of Lovo-Cel is generally favorable, with adverse events that can be managed.

### 3.6. Gene Therapy of β-Thalassemia Using LV-GLOBE

In a phase I/II clinical study (TIGET-BTHAL), a modified vector lacking the HS4 LCR element (LV-GLOBE) encoding modified β-globin β^AS3^ was used for the transduction of autologous CD34^+^ cells derived from three adults and six children with β° or β^+^ thalassemia [48]. Three out of four pediatric patients discontinued RBC transfusions, while the transfusion requirement was reduced in the adults [48]. Younger age and persistence of a higher number in the repopulating stem cells were associated with better outcomes [48].

### 3.7. Gene Therapy for Pediatric β°/β° Using Modified LV Globin

Li et al. have reported an interim analysis of a single-arm pilot trial evaluating a β-globin expression-optimized and insulator-engineered lentivirus-modified autologous HSCs in β°/β° pediatric β-thalassemic patients [49]. This study was based on the construction of a vector (BD211) containing the WT-β-globin gene as a transgene, various elements of the LCR, the enhancers of BCL11A and glycophorin A promoter to ensure erythroid-specific expression of the HBB cDNA, and a short insulator derived from foamy viruses, which reduced the genotoxicity potential of the U3 region of the LTR [49]. The distinctive properties of this vector consisted of (i) an insulator design integrated into the lentiviral vector to minimize the risk deriving from potential insertional mutagenesis, and (ii) an optimized β-globin gene expression cassette engineered to achieve physiological hemoglobin levels compared to those observed in healthy subjects [49]. Preclinical studies supported the safety and efficacy of BD211 CD34^+^ cells in NCG-X mice (triple-immunodeficient mouse with a point mutation in c-kit); particularly, these cells efficiently engrafted and differentiated into human erythroid cells within the mouse bone marrow and blood [50].

A clinical study reported the efficacy of autologous BD211 CD34^+^ cells in two female pediatric β°/β° thal patients [49]. Engraftment of genetically modified HSCs and HPCs was successful and sustained in both patients; both patients reached a condition of durable transfusion independence; both patients showed a stable vector copy number of 2–4 copies per cell [49]. Single-cell analysis showed vector inserts in 33–65% of cells, and no dominant clones were observed.

### 3.8. Gene Therapy Using Lentiviral BCL11A Short Hairpin mRNA

The Boston Children’s Hospital developed a gene therapy based on the lentiviral vector LVV BCH-BB694 encoding a microRNA-adapted short hairpin (shRNA^miR^) targeting BCL11A [51]. In preclinical studies, this vector efficiently transduced CD34^+^ cells with a high gene marking and induced HbF synthesis [51]. Thus, a phase I clinical study (NCT 03282656) evaluated the safety and efficacy of HbF synthesis induction by lentiviral gene addition with a short hairpin RNA embedded in a microRNA (shmiR) against BCL11A driven by an erythroid-specific promoter [52]. A first report on this study showed the results on the first six SCD patients enrolled, showing a significant gene marking in whole blood (VCN 0.49-1.49) 6 months after infusion, with a significant and stable induction of HbF synthesis (20–41%, median level 31%) [52]. Three severe adverse events were reported, including influenza infection, leg pain, and recurrent priapism [52]. No events of vascular occlusion, acute chest syndrome, or stroke after infusion were reported [52]. An updated analysis of this study was extended to 10 SCD patients, showing that post-transcriptional gene knockdown of BCL11A was safe, associated with stable HbF induction and significant mitigation of vascular occlusive events: in six SCD patients with frequent vascular occlusive events before treatment, between zero and one events of vascular occlusion after gene therapy were reported; in three SCD patients treated with chronic transfusion regimens before treatment, transfusion independence was reached in 2/3 cases [53]. A recent long-term follow-up of these 10 patients treated with LVV BCH-BB694 gene therapy confirmed the results previously reported [54]. Among the 10 treated patients, the patient with the lowest in vivo VCN had the lowest HbF post-infusion level of 14.1% that remained stable during the whole follow-up; a robust Hb F induction was observed in the other nine treated patients, ranging from 20% to 38.2%, F-cells ranging from 55% to 89%, and HbF content/F-cell ranging from 9.4 pg to 13.6 pg [54]. The analysis of patient and parent-reported outcomes post-treatment with LVV-BCH-BB694 supported a significant improvement in quality of life related to the decrease in vascular occlusive events and in transfusion rates [55].

A single-cell analysis on RBCs of SCD patients treated with LVV-BB694 gene therapy, compared to RBCs derived from SCD patients treated with hydroxyurea showed in the first group of patients fewer RBCs with high content of HbS that could be susceptible to sickling compared to the second group of patients; conversely, RBCs from patients treated with LVV-BB694 are more resistant to sickling at physiologic O_2_ tension compared to those from SCD patients treated with hydroxyurea [56].

Finally, whole-genome sequencing of individual HSCs/HPCs of six SCD patients undergoing BCH-BB694 gene therapy was evaluated, showing that no clonal expansions of gene-modified or un modified cells were observed; however, an increased frequency of potential driver mutations associated with clonal hematopoiesis or with myeloid neoplasms (*DNMT3A* and *EZH2* mutated clones) was observed both in genetically modified and unmodified cells, thus suggesting a positive selection of mutant clones during gene therapy [57].

## 4. Gene Editing Therapy of Beta Hemoglobinopathies

Gene editing therapy in hemoglobinopathies is a recently developed technology to modify genetic information in patients with hemoglobinopathies. This technology offers the advantage with respect to gene addition studies using lentiviral vectors to enable gene modification without the need to generate an expression lentiviral vector and introduce exogenous DNA. Two different versions of gene editing were used for therapy of hemoglobinopathies: (i) gene editing for correction of single-nucleotide mutations, such as the SCD mutation at the level of the β-globin gene; (ii) gene editing of a regulatory DNA sequence to induce HbF synthesis (SCD and β-thalassemia). Gene editing does not require the use of lentiviral vectors and is based on the introduction into patients’ cells of engineered nucleases for site-specific editing.

Gene editing techniques can be subdivided into two different groups: gene editing based on double-strand break; gene editing without a double-strand DNA break.

### 4.1. Gene Editing Based on Double-Strand Break

Several engineered nucleases for site-specific editing are available, including the CRISPR/CRISPR-associated protein 9 (Cas9) system, zinc finger nucleases, and transcription activator-like effector nucleases (Table 4). Among these various systems, the CRISPR/Cas9 system is the most frequently used in gene therapy studies for its high efficiency in gene editing in the most primitive and quiescent compartments of HSCs and HPCs.

The CRISPR/Cas9 is a two-component system based on the utilization of nucleases, allowing the generation of DNA double-strand breaks into specific DNA sequences of the genome; this system is composed of a caspase 9 (Cas9) that is driven on a specific DNA target sequence by a single-guide RNA (gRNA); the Cas9 recognizes this sequence and cleaves DNA. The cell attempts to repair double-strand breaks by using two different repair mechanisms. Non-homologous end joining repair (NHEJ) and homology-directed repair (HDR). NHEJ creates insertions and deletions (indel) at the cut site, which can inactivate the gene by disrupting the coding sequence or can alternatively increase or decrease gene expression by modifying the binding site of a transcriptional repressor or activator, respectively. However, it is difficult to drive exactly the nature of the editing process in terms of insertion or deletion, and the extent of these deletions or insertions in terms of the number of nucleotides inserted or deleted. Alternatively, the HDR system utilizes a donor DNA template sequence that directs the host genome to repair the cut site, matching the template [58]. These two repair processes differ not only in their mechanisms but also in their occurrence in different cell cycle phases: HDR-based editing is confined to the S/G2 cell cycle phases, thus limiting it to the most primitive and quiescent compartment of HSCs/HPC, while the NHEJ editing system operates in quiescent cells (Table 4).

CRISPR/Cas 9 was used as a gene editing tool in clinical studies aiming to disrupt regulatory elements, such as the erythroid enhancer of the *BCL11A* gene or the BCL11A-binding elements present in the promoter of HBG1 and HBG2 genes, or to correct the point mutation observed in SCD patients.

### 4.2. Gene Editing Without Double-Strand DNA Break

Base editing and prime editing represent two strategies to modify DNA sequences without inducing a double-strand break.

Base editing is a genome editing approach that uses components of the CRISPR systems (catalytically inactive Cas9 nickase or dead Cas9) with other enzymes (deaminases) to directly install point mutations into cellular DNA or RNA without making double-strand DNA breaks. Base editors directly convert one base or base pair into another, enabling the efficient installation of point mutations in non-dividing cells [59]. PAM (protospacer adjacent motif) is a short DNA sequence required for CRISPR-Cas editing to occur, mediating Cas enzyme binding, unwinding of DNA, and cutting of the target. PAM sequence is located immediately downstream of the target DNA sequence recognized by the guide RNA.

A base editing system was developed to correct the SCD genetic defect using a PAM nickase specifically recognizing the SCD mutation site, fused to an adenine deaminase [49]. Electroporation of this base editing product in CD34^+^ cells resulted in 80% conversion of the SCD mutation into the non-pathogenic HbG^Makassar^ variant (β6 Glu ⟶ Ala) [52]. Base edited CD34^+^ cells maintained their gene-editing in their blood progeny (44% gene-edited cells 16 weeks after transplantation in mice) [52]. This gene editing significantly inhibited RBC sickling [60]. HbG-Makassar is a naturally occurring variant that is clinically asymptomatic. Studies in transgenic mice model expressing homozygous HbG-Makassar showed a normal RBC physiology and RBC physiology comparable to WT HbA; RBCs HbGS displayed a hematological phenotype intermediate between HbAS and HbSS, supporting the use of base editing of HbS to HbG-Makassar in SCD patients [60]. Importantly, base-edited CD34^+^ β^S^ cells converted to β^Makassar^, displayed an efficient engraftment capacity in nonhuman primates, rapidly regenerating all hematopoietic lineages [61].

Several studies have shown that base editing of DNA sequences involved in the control of HbF synthesis represents an important strategy for potential therapeutic implications. Thus, the mutation of the −175 γ-globin nucleotide A to G determines a strong induction of HbF synthesis at levels higher than those elicited by Cas9 strategies targeting a BCL11A motif in the γ-globin promoter or a BCL11A erythroid enhancer [62]. Comparison with strategies using Cas9 showed that disruption of the BCL11A-binding motif at the *HBG1/HBG2* promoters elicited sustained HbF synthesis in healthy and β-thal patient HSCs/HPCs [55]. Importantly, base editing of the γ-globin gene promoter induces potent HbF synthesis without detectable off-target mutations in HSCs/HPCs [63].

The base editing may represent an efficient strategy to disrupt a DNA regulatory element, with the aim of blocking its function. In fact, a recent study by Fontana and coworkers provided evidence that multiplex base editing represents an efficient strategy to disrupt the +58K and +55 enhancer of the *BCL11A* gene, resulting in a reactivation of HbF to levels exceeding those achieved with CRISP-Cas9-induced editing, minimizing double-strand breaks and genomic rearrangements [64].

An alternative approach was proposed by Rajendiran and coworkers, who have base-edited the zinc finger domain (Znf4, ZnF5, and ZnF6) used by BCL11A to interact and repress the *HBG1* and *HBG2* promoters [54]. Base editing of ZnF4 and ZnF6 induced elevated HbF synthesis without affecting normal hematopoiesis [65].

Base editing strategy was under evaluation in clinical studies assessing the efficacy of autologous HSCs/HPCs base-edited at the level of HBG1 and HBG2 promoter sequences involved in repression of HbF synthesis in adult erythroid cells.

Prime editing is a technology of gene editing allowing the direct writing of new genetic information into a targeted DNA sequence. This technology uses a prime editing guide RNA (peg RNA) capable of identifying the target site and providing new genetic information to replace the target DNA nucleotides and a fusion protein, formed by a catalytically impaired Cas9 endonuclease fused to an engineered reverse transcriptase enzyme. Prime editing is capable of mediating targeted insertions, deletions, and base-to-base conversions without the need for double-strand breaks or donor DNA templates [66]. The prime editing methodology was recently improved through the development of prime editors exhibiting a uniquely high level of editing precision that are highly error-prone [67].

Everette et al. showed that prime editing can correct the SCD allele β^S^ to WT β^A^ at frequencies of 14–41% in HSCs/HPCs derived from patients with SCD [68]. Several weeks after transplantation into immunodeficient mice, prime-edited SCD maintained β^A^ levels and displayed engraftment rates and lineage differentiation capacities comparable to those of WT HSCs [68].

Furthermore, a recent study provided evidence that multiple editing of γ globin gene promoters by prime editing in erythroid cells induced an enhanced capacity to reactivate HbF synthesis compared to cells with individual mutations [69].

Fiumara and coworkers have compared base-editing and Cas9 in human HSCs/HPCs concerning editing efficiency, cytotoxicity, transcriptomic changes, and on-target and genome-wide genotoxicity [70]. Base editing and prime editing induced detrimental transcriptional responses that reduced editing efficiency and hematopoietic repopulation in xenotransplants and also generated double-strand breaks and genotoxic products at a lower frequency than Cas9 [70]. These findings raised concerns about the potential of genotoxicity of base editing and prime editing and suggested careful additional evaluations in view of clinical applications [70]. Cas9-mediated double-strand breaks induce large deletions at a frequency approximately 20-fold higher than base editing and prime editing [71].

### 4.3. Specific Transcriptional and Epigenetic Modulation Using Dead Caspase 9 (dCas9)

The considerable progress made in gene editing technologies has provided the rationale for the development of a technology aiming to specifically and stably modulate gene expression through epigenetic mechanisms, thus providing a tool for therapeutic interventions without altering the target DNA sequence. This approach involves CRISPR activation (CRISPRa) and CRISPR interference (CRISPRi) and requires the use of catalytically dead Cas9 (dCas9) fused to different transcriptional effectors [72,73]. The CRISPRa systems were based on transcriptional activators, while the CRISPRi systems involve transcriptional repressors. The CRISPRi represents a programmable and reversible strategy of gene silencing; in this technique, specific single guide RNAs drive dCas9 at the level of promoter regions or in proximity of start transcription sites, inhibiting RNA polymerase binding, thus blocking gene expression [74].

dCas9 may also be fused to epigenetic effectors to modulate gene expression by epigenetic mechanisms. Interestingly, a recent study showed that modulation of the methylation status of the promoter of HBG by epigenome editing may represent a potential therapeutic for β-hemoglobinopathies [75].

### 4.4. CRISPR-Cas9 Editing of BCL11A Enhancer: Studies in β-Thalassemia

A pivotal study by Ye and coworkers showed that the CRISPR-Cas9 genome-editing technology can be used to modify the *β*-gene locus using NHEJ to generate modifications suitable for therapy of patients with SCD and β-thalassemia [76]. In 2019, Wu et al. showed that Cas9/sgRNA-mediated cleavage within a GATA1 binding site at the +58 BCL11A enhancer results in disruption of this motif, reduction in BCL11A expression, and induction of fetal γ-globin [77]. Gene editing of the BCL11A erythroid enhancer appeared to represent an important strategy to induce therapeutically relevant high levels of HbF synthesis in SCD and β-thalassemia [77].

A recent study clarified the mechanism through which CASPR editing of the BCL11A enhancer causes repression of BCL11A expression and induction of HbF synthesis [78]. The E+58 enhancer maintains chromatin insulation and prevents epigenetic silencing of the *BCL11A* gene through two key functions: serving as a transcription start site for production of enhancer RNA (eRNA promotes enhancer-promoter looping); acting as a site of interaction for NIBPL (Nipped-B-Like-protein) and cohesion complex proteins for chromatin loop formation [78]. CRISPR editing of the BCL11A +58 enhancer disrupts both its functions, causing a destabilization of chromatin configuration at the promoter level, impaired epigenetic chromatin insulation, and epigenetic silencing of *BCL11A* [78].

A pilot clinical trial provided the first evidence in favor of the safety and efficacy of CRISPR/Cas9 gene editing of the BCL11A erythroid promoter in SCD and β-thalassemia patients. The report on the first two patients enrolled in the CLIMB-THAL 111 and CLIMB-SCD 122 studies, one β°/β^+^ thal and one β^S^/β^S^ SCD patient, showed a rapid rise in HbF and Hb levels after infusion of autologous CD34^+^ gene-edited cells, a pancellular distribution of HbF at the level of RBCs, and the permanence of gene-edited cells in peripheral blood [79]. Both thalassemic and SCD patients became transfusion-independent, and in the patient with SCD, no vaso-occlusive events occurred [79].

The results observed in the phase III CLIMB-THAL 111 study were recently published; in this study, 52 patients with β°/β°, β°/β^+^, and β°/β°^like^ transfusion-dependent disease received autologous CD34^+^ cells. Gene-edited with CRISPR-Cas9 at the level of BCL-11A erythroid enhancer (with the commercial name of Exaglamblogene, Exa-Cel) [79]. 91% of these patients become transfusion-independent, with a mean Hb level of 13.1 g/dL and a mean HbF level of 11.9 g/dL, distributed in 94% of RBCs; only 9% of patients did not reach transfusion independence but decreased their transfusion rates [80]. Allelic editing remained stable during the first 6 months after gene therapy. The safety profile of Exa-Cel-treated patients was consistent with busulfan conditioning [81]. An updated analysis extended to 54 β-thal patients confirmed transfusion-independence in 94% of patients receiving Exa-Cel, with sustained increases in HbF and total Hb for up to 5 years of follow-up [82]. An analysis of these patients with a follow-up extended up to 6 years showed that Exa-Cel-based gene therapy not only induced transfusion independence but also led to a normalization of iron metabolism: in fact, after Exa-Cel treatment, iron was successfully removed by iron-replating therapy [82]. This observation further supports a curative effect of Exa-Cel in β-thal patients [82].

A similar approach was used by Fu et al., who initially reported the results on two β-thal β°/β° pediatric patients, transfusion-dependent, treated with autologous HSCs/HPCs BCL11A enhancer-edited in the context of the phase I/II trial NCT 04211480 [83]. Both patients achieved transfusion independence, and their Hb increased from 8.0 to 10.8 g/dL at screening to 15.0 and 14.0 g/dL, with >85% editing persistence in bone marrow cells [83]. An updated report on this study displayed the results observed in 10 β-thal patients (5 β°/β°, 4 β^+^/β°, and 1 β^+^/β^+^), showing a sustained rise in HbF and F-cells, reaching values of 98–99% [84]. Adverse events were related to busulfan conditioning. A more recent update to 15 β-thal patients (8 β°/β°, 4 β^+^/β°, and 3 β^+^/β^+^) confirmed and extended the results observed in previous studies [85].

### 4.5. CRISPR-Cas9 Editing of BCL11A Enhancer: Studies in Sickle Cell Disease

Exa-Cel gene therapy was also investigated in SCD patients. The phase III CLIMB SCD-121 clinical trial explored the safety and therapeutic efficacy of CD34^+^ autologous cells gene-edited using Exa-Cel in 44 transfusion-dependent SCD patients [86]. After Exa-Cel gene therapy, the large majority of treated patients were free from hospitalizations for severe vaso-occlusive crises; only six severe vaso-occlusive events were observed in patients whose increase in total Hb and HbF levels was similar to that observed in patients without vaso-occlusive crises [86]. Total Hb levels increased from <9 g/dL to 12.5 g/dL six months after the start of therapy, with 44.5% of HbF; all patients displayed a decrease in hemolysis and became transfusion independent. Adverse events were those expected for patients receiving conditioning with busulfan and autologous HSC transplantation [86]. An updated analysis of the results of this trial encompassing 46 SCD patients with a more extended follow-up showed that elimination of vascular occlusive events was observed in 90% of patients receiving Exa-Cel, with a marked increase in total Hb (>12 g/dL) and HbF (>40%) [87]. In CLIMB SCD-121 and CLIMB-131 combined, 100% of patients were free of severe vascular occlusive crises for ≥12 consecutive months, and 100% were free from inpatient hospitalization for severe occlusive crises [88]. These observations support a curative effect in SCD patients undergoing Exa-Cel-based gene therapy [88].

The long-term effects of Exa-Cel-based gene therapy observed in the CLIMB SCD-121 study are under evaluation in the context of the CLIMB 131 study. The analysis of the quality of life of these patients showed a remarkable improvement in quality of life, both for adolescent and adult patients [89].

In the CLIMB THAL-141 and CLIMB SCD-151 studies, Exa-Cel was infused following busulfan myeloablation to patients aged 2–11 years with a history of transfusion-dependent β-thalassemia or SCD with ≥2 severe occlusive events per year for 2 years before screening [90]. Efficacy and safety data for the first 26 patients (13 SCD and 13 β-thal) are consistent with the data observed in patients aged ≥12 years, with clear signs of clinical benefit and with a safety profile consistent with busulfan conditioning and autologous HSCT [90].

### 4.6. Gene Editing of γ-Globin Gene Promoters Using CRISPR-Cas9 Technology

The expression of human γ-globin genes is finely regulated during development by multiple mechanisms. The expression of *HBG1* and *HBG2* genes in human erythroid cells is almost completely repressed through efficient transcriptional and post-transcriptional mechanisms [91]. Some gene therapy studies aimed to reactivate HbF synthesis in adult erythroid cells by interfering with the genetic mechanisms that mediate the transcriptional repression of *HBG1* and *HBG2*. In this context, the two main targets are represented by the binding sites of the transcriptional repressors BCL11A and LRF present in the promoter region of *HBG1* and *HBG2* genes. In a recent study, Wongbrisuth et al. comparatively evaluated the effect of the disruption of LRF or BCL11A binding sites of the γ-globin gene promoter by CRISPR/Cas9 gene editing in CD34^+^ cells isolated from healthy or β°/HbE individuals: both disruptions similarly increased HbF synthesis, without affecting erythroid differentiation and with minimal off-target effects [92].

Some studies used CRISPR-Cas9 to edit sequences of *HBG1* and *HBG2* genes involved in gene repression. In this context, an initial study by Traxler and coworkers showed that the introduction of a mutation in the −102 to −114 *HBG1* promoter by CRISPR-Cas9 enabled the production of erythroid cells with an increased HbF synthesis capacity [93]. Disruption of the *HBG1/HBG2* gene promoter motif that is bound by BCL11A using CRISPR/Cas9-mediated gene editing in CD34^+^ cells resulted in the generation of erythroid cells exhibiting a consistently increased HbF synthesis [94].

These studies have provided the preclinical basis for a clinical trial involving gene editing of *HBG1* and *HBG2* gene promoters using CRISPR-Cas9 technology. To this end, Sharma et al., in the first set of experiments, defined the guide RNA for optimal targeting of *HBG1* and *HBG2* promoters; then, using gRNA-68 selected for clinical studies, edited CD34^+^ cells isolated from SCD patients [95]. The editing product was defined as OTQ923. Three SCD patients were treated with OTQ923 and displayed a significant increase in HbF levels, ranging from 19% to 25%; all three patients had a significant improvement in their hematological levels but still displayed signs of mild hemolysis; all three treated patients had at least one episode of vaso-occlusive crises [95]. These findings suggest that the levels of HbF synthesis observed after infusion of gen-edited autologous HSCs/HPCs were not sufficient to inhibit HbS polymerization completely [95].

### 4.7. Base Editing of BCL11A Binding Site in γ-Globin Gene Promoter

BEAM-101 is a gene therapy for hemoglobinopathies that uses adenine base editors to introduce single-base changes (A to G substitutions) in the γ-globin genes *HBG1* and *HBG2* in patients’ HSCs/HPCs ex vivo. These changes disrupt the binding of BCL11A repressor, increasing the expression of fetal hemoglobin production. Preclinical studies showed that base editing potently induced HbF synthesis (>60%) and proportionately reduced HbS (<40%). Two recent studies reported the preliminary results observed in the first six SCD patients enrolled in the phase I/II clinical study BEACON involving autologous stem cells gene-edited with BEAM-101 [96]. These results supported the efficacy of this treatment in terms of an increase in HbF levels and inhibition of vascular occlusive events [96,97]. One patient died during treatment of respiratory insufficiency related to busulfan conditioning [96,97].

Beam Therapeutics recently updated the results of the BEACON study, reporting the results on the first 17 enrolled patients [98]. The presented data showed that all 17 patients achieved HbF levels over 60% and HbS levels below 40%, with these effects lasting for up to 15 months; lack of vaso-occlusive crises post-treatment was observed in all patients; markers of hemolysis normalized or improved, and erythropoietin levels decreased, indicating better oxygen delivery; the safety profile remained consistent with the expected results of busulfan conditioning [98]. A very recent update, extended to the first 26 patients, confirmed the results reported in previous reports and showed that in all treated patients a robust HbF synthesis was observed, with total Hb levels reaching 15.6 g/dL by 6 months after infusion of engineered HSCs; peripheral blood cell editing was high, with >72% of cells gene-edited 6 months after infusion of gene-edited HSCs [99]. It is important to note that the BEAM-101 treatment process is associated with high editing efficiency and rapid neutrophil and platelet engraftment, showing the potential to minimize hospitalization [99].

Wang and coworkers reported the development of a transformer base editor (tBE) system involving a cleavable deoxycytidine inhibitor (dCI) that induces efficient editing with only background levels of genome-wide and transcriptome-wide off-target mutations [100]. After being produced, the tBE remains inactive at off-target sites with the fusion of a cleavable dCI, therefore eliminating unintended mutations [100]. Correspondence Sequence Therapeutics (CST) used tBE for the editing of the γ-globin *HBG1* and *HBG2* promoters (CS-101 product). Preclinical studies showed that CS-101-mediated editing of *HBG1* and *HBG2* promoters induced robust HbF synthesis, without causing adverse events on the engraftment or differentiation of HSCs in mice after transplantation [101]. The preliminary results obtained in the first 11 β-thal patients transplanted with autologous HSCs gene-edited with CS-101 showed clinically significant increases in both total Hb and HbF, prompt and durable engraftment, and therapeutic benefits (transfusion independence) [102,103]. The treatment elicited a rapid increase in HbF levels with pancellular distribution and stable editing efficiency [102,103]. Very recently, Chen et al. reported at the ASH Meeting 2025 the results on the first 14 β-thalassemic patients treated with CS-101, showing transfusion-independence in 12/14 patients, with HbF levels above 12 g/dL from month 4 onwards; the allele base editing efficiency in bone marrow and peripheral blood nucleated cells remained stable over time [104].

### 4.8. Cas12 Editing of BCL11A Binding Site in γ-Globin Genes Promoter

Another study of gene editing of the *HGB1* and *HBG2* promoters utilized the CRISPR-Cas12 technology. There are several remarkable differences between Cas-12 and Cas-9; the most remarkable difference is that Cas-12a possesses a single nuclease domain and intrinsic RNA processing activity, allowing multigene editing of RNA transcripts and resulting in staggered DNA ends, promoting HDR instead of NHEJ [105]. The CRISPR-Cas12a system was used to edit the distal CCAAT-box region of the *HBG1* and *HBG2* promoters, resulting in a high editing rate of these DNA sites, associated with the induction of elevated HbF synthesis in the erythroid progeny (about 40%) [106]. Importantly, gene-edited HSCs efficiently repopulate the hematopoietic system, and no off-target editing was observed [97]. Using this approach, the EDIT-301 gene editing product was developed. Using this product, two ongoing clinical trials were initiated. The RUBY trial is evaluating the safety and efficacy of EDIT-301 in adult and adolescent participants with severe SCD, while the EdiThal trial evaluated the safety and efficacy of EDIT-301 in transfusion-dependent β-thalassemia. The initial results of the first seven and two patients enrolled in the RUBY and EdiThal studies were reported [107]. Successful engraftment was observed in all patients: in SCD, a rapid and sustained normalization of Hb 4 months after infusion was observed, associated with a marked increase in HbF synthesis, associated with resolution of vaso-occlusive events and normalization of the markers of hemolysis; in the two β-thalassemic patients, rapid improvements of hematological parameters were observed, associated with development of transfusion-independence [107]. The NCT 03663760 study will evaluate the long-term safety and efficacy of EDIT-301 in SCD and β-thalassemia patients.

An updated report of the RUBY trial included the results on 21 SCD patients supporting the efficacy of EDIT-301 (renamed as Reni-Cel): total Hb was 14.2 ± 2 g/dL at month 6 and was maintained up to last follow-up; mean percentage of HbF was 48.2% at month 6 and was maintained at >40% through last follow-up; markers of hemolysis improved or normalized; all patients were vascular event-free [108]. The safety profile was consistent with myeloablative conditioning with busulfan [99]. A very recent report updated the results of the RUBY trial on 32 SCD patients, with 31/32 patients reaching a condition of absence of vascular occlusive events after Reni-Cel infusion and with all patients reaching a normalization of Hb levels (13.8 ± 1.8 g/dL), with >40% of HbF [108].

An updated analysis on seven β-thalassemic patients treated in the context of the Edit-Thal trial was recently reported [109]. In all treated patients, HbF concentration increased early and was 11.3 ± 1.7 g/dL by month 6; the percentage of F cells was >99%; all seven patients reached a transfusion independence status [109]. A high level of gene editing (>75%) was observed in PB cells and in CD34^+^ cells [109]. The safety profile was consistent with myeloablative conditioning with busulfan.

### 4.9. Gene Correction Studies Using CRISPR-Cas9 Gene Editing

Uchida et al. reported an efficient gene correction strategy for the SCD mutation in the β-globin gene with electroporation-mediated delivery of editing tools, achieving therapeutic-level correction at the DNA level (about 30%) and the protein level (about 80%) [110]. This virus-free gene correction system, used to correct β^S^ mutation, utilizes SCD mutation-targeting guide RNA, Cas9 mRNA/protein, and donor ssDNA encoding the normal β-globin sequence [110]. The gene-edited CD34^+^ cells with correction to the normal β-globin gene sequence were engraftable in mouse and primate models [110].

The Cedar phase I/II clinical study evaluated the efficacy of autologous HSCs with correction of a single nucleotide mutation (GPH 101) to convert HbS to HbA for treating severe SCD [102]. Thus, GPH 101 is an investigational, autologous, HSC drug product designed to correct the SCD mutation in the β-globin gene ex vivo using a high-fidelity Cas9 paired with AAV6 (adenovirus-associated virus type 6), efficiently harnessing the natural HDR pathway [111]. In preclinical studies, the β-globin gene in SCD donor HSCs resulted in ≥60% gene-corrected alleles in vitro with minimal off-target effects [111].

In the phase I/II Cedar trial for HSC gene-correction therapy in SCD with GPH 101, poor engraftment of HDR-edited CD34^+^ cells was observed. In the first treated patients, 2.3% editing in BM cells was detected 26 weeks post-gene therapy; however, blood cell recovery was delayed since platelet and RBC transfusions were required until 26 weeks and 38 weeks post-gene therapy, respectively [104]. This finding implies the absolute need for an improvement in the engraftment capacity of HDR-edited CD34^+^ cells for HSC gene-correction therapy for SCD. Clinically, the patients showed improvement in the quality of life with absent vascular occlusive events [112].

The reduced engraftment capacity of HDR-edited HSCs was supported by an experimental study in rhesus macaques [113]. Thus, Lee et al. have co-infused HSCs transduced with a barcoded GFP-expressing lentiviral vector and HDR-edited at the CD33 locus. CRISPR/HDR-edited cells showed a two-log decrease, 2 months following transplantation, in comparison to minimal loss of lentivirus-transduced cells long term [113]. Furthermore, HDR long-term clonality was oligoclonal in contrast to highly polyclonal lentivirus-transduced HSCs [113]. These observations suggest marked differences in the impact of genetic modification approaches on HSCs.

### 4.10. Zinc Finger Nuclease-Mediated Gene Editing of BCL11A Erythroid Enhancer in HSCs

BIVV003 is a gene-edited autologous cell therapy in which HSCs are genetically modified with mRNA encoding zinc finger nuclease (ZNF) that targets and disrupts a specific regulatory GATA motif present in the *BCL11A* erythroid-specific enhancer [114]. Zinc finger proteins combined with the nuclease domain of the restriction endonuclease Fok1 create double-strand breaks at precisely defined genomic locations. Repair of the double-strand breaks via NHEJ or MMEJ results in target sequence disruption [114]. ZNF-mediated gene editing of the BCL11A erythroid-specific enhancer markedly reactivates HbF synthesis in erythroid cells without affecting in vivo engraftment of gene-edited HSCs [114]. Preliminary results obtained in the context of the PRECIZN-1 phase I–II study in seven SCD patients showed that BIVV003 was well-tolerated and elicited an increased total Hb and HbF levels, associated with absent vaso-occlusive events [114].

## 5. Gene Therapy of β-Hemoglobinopathies Through In Vivo Gene Editing

As discussed above, the various approaches used for ex vivo gene therapy are very complex and expensive. The in vivo gene editing and gene therapy through intravenous or intraosseous delivery of gene therapy vectors could considerably simplify the manufacturing process, to overcome the numerous challenges associated with the manufacturing of gene therapy products required for ex vivo HSCs/HPCs engineering, and to reduce the costs of the whole procedure.

Various preclinical studies have supported the therapeutic efficacy of in vivo gene editing. Li et al. reported a gene therapy approach for SCD based on gene editing of the β^S^ globin gene in HSCs in vivo by infusion of a prime editing vector in mouse bone marrow [115]. Using this approach, Le et al. reported the achievement of therapeutic levels of β-globin gene correction in mouse HSCs in vivo using a prime editing vector administered intravenously in mice [115]. In this study, the prime vector was represented by an adenoviral vector with high affinity for CD46, a membrane receptor expressed on HSCs [115]. The prime vector was infused into mice with HSCs/HPCs mobilized using G-CSF/Plerixafor. With a single infusion of adenovirus carrying prime editors, about 40% of β^S^ alleles were corrected and replaced by β-WT [98]. Importantly, this level of gene correction was maintained in secondary transplants, thus supporting the efficacious gene editing of repopulating HSCs [115]. The procedure of in vivo gene editing using this delivery machinery may be simplified, replacing G-CSF as a mobilizing agent with WU-106, an inhibitor of integrin α4β1, obtaining a rapid and efficient mobilization of HSCs [116].

A similar approach using an adenoviral vector expressing an all-in-one adenine base editor to convert the β^S^ mutation into the benign HbG^Makassar^ variant was used for efficient ex vivo and in vivo correction of SCD mutation [109]. The in vivo treated animals demonstrated correction of disease features without significant side effects [117].

Other studies have adopted lipid nanoparticles as an in vivo delivery system alternative to viruses to vehiculate gene-editing machinery. Thus, Breda et al. have developed a CD117/LNP-messenger RNA, a lipid nanoparticle (LPN) that encapsulates mRNA and is targeted to stem cell receptor (CD117) on HSCs [118]. Delivery of the anti-human CD117/LNP-based editing system elicited a nearly complete correction of SCD mutation in hematopoietic cells [118]. Using the same anti-CD117/LNP delivery system, ex vivo delivery of adenine base editors to SCD HSCs/HPCs resulted in an efficient (88%) conversion to HbG^Makassar^ with up to 91.7% increase in HBG^Makassar^ protein and a nearly complete absence of sickled RBCs in mature erythroid elements [118].

Lian et al. have explored several bone marrow-homing nanoparticles and showed that they deliver mRNA to a broad group of hematopoietic cell types, including HSCs and HPCs [119]. Using these LPR-nanoparticles, CRISPR/Cas9 and base editing were achieved in a mouse model expressing human sickle cell disease phenotypes for potential HbF reactivation and conversion from sickle to non-sickle alleles [119].

Xu and coworkers reported the development of an efficient LPN-based delivery system for in vivo gene editing of the *BCL11A* enhancer and of the HBG promoter [111]. This system uses antibody-free targeted nanoparticles (LPNs) for mRNA delivery to HSCs in vivo, allowing efficient base editing of the *HBG1* and *HBG2* promoters in human HSCs [120]. The system uses ABE8e, an adenine base editor with an active, efficient adenine conversion, and showed highly efficient on-target adenine base edits at the level of regulatory regions of *BCL11A* and *HBG* genes [121]. Delivery of ABE8e/sg RNA with optimized LPNs (containing lipid-168 possessing enhanced delivery efficiency in vivo) achieved efficient in vivo base editing of the *HBG1* and *HBG2* promoters in β-thal HSCs that engrafted into immunodeficient mice, showing restored globin chain balance in erythroid cells [121].

Milani et al. recently reported an interesting observation related to the high trafficking of HSCs/HPCs observed in newborn mice as well as in humans after birth, thus showing an ontogenic window suitable for in vivo gene therapy studies [122]. The system of in vivo gene editing of HSCs is also optimized through the use of lentiviruses engineered for in vivo administration and tissue-targeted expression [123]. Using this system, the efficient in vivo gene transfer into long-term HSCs in newborn mice was reported [122]. The efficacy of this in vivo strategy of HSC gene therapy was tested in the context of mouse models of various hereditary HSC defects [122].

Other studies showed the highly efficient transduction in vivo of HSCs using adenovirus-associated virus serotype 6 (AAV6) vectors as in vivo delivery systems [124]. In situ gene editing of HSCs can be achieved via AAV6-delivered CRISPR guide RNA [125]. The efficacy of this in vivo gene editing system was shown in an SCD mouse model treated with AAV6 delivering a Cas9 editing system enabling induction of therapeutic levels of HbF [125].

A recent study explored the use of extracellular vesicles (EVs, membrane-limited particles secreted by the cells) as a delivery system for in vivo gene therapy [126]. Bhoorasingh et al. used EVs engineered by loading Cas9 RNP complexes and by expressing kit ligand for efficient targeting of HSCs [126]. These EVs have an efficient targeting of c-kit expressing cells and may vehiculate CRISPR-Cas9 RNP complexes to HSCs/HPCs and may be suitable for in vivo gene therapy of SCD [126].

Other studies used a new technology of gene editing developed by Tessera Therapeutics, based on RNA gene writers that use an RNA template and a protein to make changes to DNA without making double-strand breaks. This system involves an RNA template and an RNA encoding for the writer protein packaged into LNPs for delivery into cells; into the cells, the mRNA is translated into the writer protein, which then binds to the RNA template. This complex travels to the genome, nicks one strand of the DNA, and uses the RNA template to write new DNA into the genome [127]. Using LPR-nanoparticles targeting HSCs and vehiculating RNA Gene Writers designed to make the wild-type correction or the HB^Makassar^ correction of β^S^, an efficient in vivo editing of HbS to HbA or to Hb^Makassar^ was shown (in primate models, an in vivo editing efficiency of 20–24% was observed) [128].

In conclusion, in vivo delivery of gene therapy through viral vectors or nanoparticles offers some consistent advantages compared to ex vivo gene therapy, including the opportunity to avoid all the complex ex vivo procedures required for the collection and isolation of HSCs/HPCs, their ex vivo culture, genetic engineering, and infusion to patients, thus simplifying the clinical gene therapy protocol. Furthermore, in vivo gene therapy approaches offer the opportunity to broaden access to this therapy. However, the introduction of in vivo delivery of gene editing agents has to face many challenges, such as achieving therapeutically efficient amounts of editing agents to the target cells; avoiding base-editing at the level of nontarget cells and tissues; and reducing the immunogenicity of the vector (particularly for adenovirus-based vectors) [129].

## 6. Affordability of Gene Therapies for Hemoglobinopathies

The approval of Exa-Cel, Beta-Cel, and Lov-Cel as a potentially curative agent for SCD and β-thalassemia represented a milestone in the field and one of the most remarkable successes of modern medicine. However, these treatments based on ex vivo gene therapy are resource-intensive and not suited for the treatment of a large number of patients. Various factors represent major challenges to the diffusion of these therapies. First, among them, the high cost of these therapies, estimated at USD 2.2 million per patient for Exa-Cel, and USD 3.1 million for Lov-Cel. A second limiting factor is that these treatments are feasible only in highly specialized medical centers, and skilled personnel and substantial infrastructures are required throughout the whole treatment. The obvious consequence is that market-driven pricing and the need for adequate healthcare infrastructures required for delivering these therapies will completely limit gene therapy treatments in the developing world.

These considerations have animated a consistent debate to critically evaluate the affordability and accessibility of gene therapy for hemoglobinopathies. In this context, an important element of discussion is the comparison of transplantation-based studies and gene therapy studies in terms of costs and patients’ eligibility.

Allogeneic hematopoietic cell transplantation (allo-HSCT) is a curative therapy in SCD; however, until recently, it was constrained by limited donor availability, by the risk of graft versus host disease (GVHD), graft rejection, and death [130]. However, recent important advances have considerably mitigated these barriers. In fact, recent studies showed that haploidentical bone marrow transplant in SCD patients after non-myeloablative conditioning regimens was associated with a high rate of engraftment (88% to 95%), 2-year overall survival ranging from 94% to 95%, acute GVHD ranging from 4% to 10%, chronic GVHD ranging from 10% to 22%, and a transplantation-related death rate of 5–7% [8,9]. The use of allo-HSCT in β-thalassemia is more consolidated, and retrospective analysis of long-term survival and of late effects showed a non-relapse mortality of about 10% (mostly observed during the first year after allo-HSCT) and an overall survival of 81.4% after 39 years, as well as a cumulative incidence of secondary solid cancers of 16.4% [131,132]. The incidence of secondary solid cancers was fourfold to sixfold higher than for hematopoietic cell donors and non-transplant patients [131,132].

At the moment, several considerations related to the cost (the cost of allo-HSCT is about five-sixfold lower than the cost of approved gene therapies of hemoglobinopathies) and to the long-term effects (that are well known for allo-HSCT, but only in part established for gene therapy treatments) favor allo-HSCT over gene therapy treatments [130].

These recommendations are important for an optimal selection of patients with hemoglobinopathies for gene therapy studies. A decision-making algorithm was developed by a group of experts in hemoglobinopathies and/or transplantation of the EHA and EBMT who discussed the selection of SCD patients for gene therapy/editing studies [126]. The team of experts has presented a document proposing to select the access of gene therapy to SCD patients in good clinical condition with no HLA-matched donor available, without irreversible severe organ impairment, and able to tolerate myeloablative conditioning, since these patients are likely to obtain the most benefit with the lowest risk [126]. This approach is fully justified by both the limited clinical experience so far accumulated during clinical studies and the limited availability of gene therapy [133].

There is no demonstration of the feasibility or safety of HSC-based gene therapy after failed allo-HSCT. Therefore, the decision whether to pursue an autologous gene therapy after a previous unsuccessful allo-HSCT may need to be made on a case-by-case basis [134].

Given the high cost and the elevated technological and infrastructural requirements, it is not surprising that multiple barriers compound challenges in gene therapy access, exacerbating existing health inequities. These include the existence of some major barriers at financial, geographic, and sociocultural levels, all of which will contribute to determining who receives these therapies first or not at all [128]. Kelkar et al., considering all these factors, proposed eligibility criteria for SCD and β-thalassemia patients for gene therapy treatments. These criteria are broader than the eligibility criteria for clinical trials but narrower than the FDA approval criteria. Thus, fear prioritization criteria are proposed for SCD and transfusion-dependent β-thalassemia patients for gene therapy studies. (i) Proportional equality: patients are prioritized in a 3:1 ratio of SCD to β-thalassemia; (ii) modified sickest first: patients with impending organ failure; (iii) lack of alternative therapy: patients without a matched donor for allo-HSCT; (iv) lottery: random sequencing of multiple patients within the same priority category [135].

## 7. Conclusions

Three approaches have been adopted for gene therapy of β-hemoglobinopathies: (i) lentiviral vector-based approaches supporting a gene addition strategy using integrating lentiviruses to introduce ex vivo into patient’s HSCs a copy of a β-globin coding sequence together with the DNA machinery required to support its high expression in erythroid cells; (ii) nuclease-based approaches supporting a gene editing strategy aiming to directly target the β-globin gene regulatory elements and consequently to reactivate HbF synthesis at therapeutic levels; (iii) base-editing approaches allowing the insertion of point mutations at level of a specific sequence in a locus, without the need for double-strand breaks and for an exogenous DNA template. Currently, three products of clinical HSC-based gene therapy for β-hemoglobinopathies have been approved by federal regulatory agencies. Thus, Betibeglogene autotemcel (a synonym of Lovotibeglogene, with the commercial name of ZYNTEGLO), based on β^A^-T87Q globin gene addition via lentivirus, was approved by EMA in 2019 and by FDA in 2022 for transfusion-dependent β-thalassemia. Lovotibeglogene autotemcel (with the commercial name of LYFGENIA), based on β^A^-T87Q globin gene addition via lentivirus, was approved by the FDA in 2023 for SCD. Exagamglogene autotemcel, based on CRISPR-Cas9 gene editing of *BCL11A* enhancer via electroporation (with the commercial name of CASGEVY)*,* was approved by EMA in 2023 and FDA in 2023/2024 for both SCD and transfusion-dependent β-thalassemia [136]. The three gene therapy products approved for SCD and β-thal treatment support a possible curative effect and represent some of the most remarkable successes of modern medicine.

Although these treatments may offer a curative approach for the therapy of SCD and β-thalassemia, they face numerous challenges due to their high costs, the complexity of manufacturing the therapeutic agents, and some safety concerns.

Concerning the safety concerns, it has to be emphasized that the safety profile of lentivirus and genome editing-based therapies is consistent with myeloablative conditioning and autologous HSCT. In this context, myeloablative conditioning represents an important limitation of current gene therapies since it can result in both severe acute and chronic toxicities. Several gene therapy studies of β-hemoglobinopathies have suggested that myeloablative conditioning is required to maximize engraftment of genetically engineered CD34^+^ cells. In the three approved gene therapy studies, fully myeloablative conditioning with busulfan was used. In the study HGB-206 of lentiviral-mediated β^T87Q^-globin transduction, the first seven patients received a busulfan conditioning regimen with a busulfan exposure target area under the curve (AUC) 65–74 mg*h/L and a cell dose of ≥1.5 × 10^6^ CD34^+^ cells/kg; a durable expression of the transgene was observed but suboptimal HBA^T87Q^ concentration of 0.51 to 1.17 g/dL were observed, requiring an adjustment of busulfan conditioning, with increased target AUC to 82 mg*h/L in group B and C [44]. In the HGB-204, -205, -207, and -212 trials, transfusion-dependent β-thalassemia patients received a myeloablative conditioning with a busulfan dose of HGB-207 and -212 with a busulfan AUC of 59–82 mg*h/L [44]. In the studies of examglogene-based gene therapy of SCD and β-thal patients, a myeloablative busulfan-based regimen was used with a busulfan AUC dose of 74–90 mg*h/L [79,84]. Also, other studies of gene editing of *HBG1* and *HBG2* promoters have incorporated myeloablative busulfan conditioning into their designs [91].

In the TIGET-Bthal trial, a reduced toxicity myeloablative regimen with treosulfan and thiotepa was used, and transfusion independence was reached only in a part of the treated patients [48]. Furthermore, in a small phase I trial carried out with a lentiglobin vector (TNS9.3.55), the patients received a reduced-intensity busulfan conditioning regimen; low but stable hematopoietic gene marking was observed, and patients did not reach transfusion independence [137]. Non-myeloablative conditioning regimens may significantly reduce the risk of these acute and chronic toxicities, may allow a wider patient eligibility, and may lower resource use, reducing the need for extensive hospital care. In this context, reduced-intensity conditioning was adopted in a few gene therapy studies of β-hemoglobinopathies, supporting that non-myeloablative conditioning can achieve durable stem cell engraftment [42]. The study of Grimley et al et al showed that the use of reduced-intensity melphalan conditioning instead of myeloablative busulfan in seven SCD patients undergoing autologous stem cell transplantation with CD34^+^ cells transduced with a lentivirus vector inducing enforced expression of γ-globin G16D decreased the duration of thrombocytopenia and neutropenia and accelerated neutrophil and platelet engraftment and reduced the time of hospitalization after transplantation compared to what observed in the studies with currently approved gene therapy products [40]. In this study, after the treatment of the first two patients, an improved strategy of HSCs/HPCs mobilization and collection was adopted with multiple collections; furthermore, the melphalan dose was adjusted according to glomerular filtration [42]. The success of reduced-intensity conditioning in this study is seemingly related to the more potent anti-sickling efficacy of γ^G16D^ than β^T87Q,^ since patients with β^T87Q^ levels in the HGB-206 study, similar to those observed for γ^G16D^ in the study of Grimley et al., had suboptimal clinical benefit [42]. Although the results are encouraging, observations on a large number of patients and on different gene therapy vectors are required to assess the effect of reduced-intensity conditioning compared to myeloablative conditioning.

Another important issue of safety is related to the risk of secondary hematological malignancies, which are complex in their multifactorial origin, not related only to the risk of insertional mutagenesis. The risk of insertional mutagenesis was related to the studies involving the use of integrating lentiviral vectors. Third-generation lentiviral-based vectors, as well as those used in Beta-Cel and Lov-Cel therapies, have been engineered to be replication-incompetent and self-inactivating, thus reducing their risk of insertional mutagenesis. No events of insertional oncogenesis have been observed in β-thal patients treated with Beta-Cel; however, two SCD patients who received Lov-Cel developed AML at 3 and 5.5 years after infusion did not show insertional oncogenesis; in one patient, leukemic cells contained the BB305 lentiviral vector [138,139]. The emergence of myeloid malignancy in gene therapy recipients with SCD is likely related to regeneration stress induced by the gene therapy procedure, triggering rapid proliferation of clones with driver mutations. These clones are related to clonal hematopoiesis, characterized by the presence of somatic mutations in the peripheral blood with a variant allele frequency ≥2% in genes associated with hematologic malignancies. Both patients with SCD [140,141] and β-thalassemia [142] display a significantly increased rate of clonal hematopoiesis compared to age-matched healthy individuals. Clonal hematopoiesis is increasingly recognized in SCD patients at younger ages than in the general population, potentially due to chronic inflammation, erythropoietic stress, and exposure to cytotoxic therapies [142,143]. Particularly, patients with CH-related mutations *TP53* and *PPM1D* may have an increased risk to develop leukemia post-transplantation [142,143].

An elevated mutation rate observed in some patients with SCD and some pressure on HSCs containing pre-existing driver mutations represent mechanisms that could increase leukemia risk in patients undergoing gene therapy for SCD and β-thalassemia [54]. A non-myeloablative conditioning regimen also has the advantage of reducing the risk of positive selection of pre-existing clonal hematopoiesis-associated driver mutations.

Another limiting factor emerging from gene therapy studies is that a fully curative effect is not frequently observed in patients most severely affected by β-thalassemia. This suggests that the efficacy of gene therapy treatments could be obtained by achieving higher levels of therapeutic Hb levels. In this context, recent studies have provided evidence that gene editing of both the *BCL11A* +58 and +55 enhancers elicited a more efficacious stimulation of HbF synthesis than a single gene editing [61,144,145]. In this setting of double editing strategies, several strategies reduced the risk of genome aberrations caused by CRISPR-Cas9-mediated double strand breaks, such as the use of base-editing and not CRISPR-Cas9 editing, the omission of ex vivo culture of gene-edited HSCs/HPCs avoiding the deleterious effects of cellular proliferation stimulated by ex vivo culture [146] and the conditioning procedure (nonprogrammed long deletions were disfavored in engrafting cells in animals conditioned by a CD45 antibody drug conjugate compared to animals conditioned using busulfan) [146]. A recent study suggested a new strategy to induce pronounced reactivation of HbF synthesis in adult erythroid cells through linear recruitment of the normally distal strong HBB enhancer to HBG through deletion or inversion of intervening DNA sequences [147]. Another recent study showed that the gene editing approach may also be extended to a precise correction of some of the most prevalent and severe β°-thalassemia-causing mutations in the β-globin-encoding HBB gene, including CD39 and IVS2.1 [148]. Gene-edited HSCs/HPCs display improved β-globin production in their erythroid progeny and correction of β-thalassemic phenotype [148].

CRISPR-Cas9 editing and lentiviral transduction have shown consistent clinical benefits, but it remains unclear which approach is superior. Alternatively, base editing also showed promising results and could reduce the risks of genotoxicity. A recent study compared three different gene therapy approaches, such as CRISPR-Cas9 editing of the *BCL11A* enhancer to reactivate HbF, lentiviral anti-sickling β^T87Q^ gene addition, and base editing converting sickle β-globin to β^Makassar^ globin in an immunodeficient mouse model [138]. All three gene-editing methods showed therapeutic potential; however, base editing and lentiviral transduction showed superior outcomes over CRISPR-Cas9-mediated editing in a competitive murine transplantation model [149].

## Figures and Tables

**Table 1:** Main steps required for gene therapy of SCD and β-thalassemic patients with ex vivo engineered autologous HSCs/HPCs.3. Gene therapy studies based on gene addition using lentiviral vectors.

Initial evaluation: patient selection and preparation
HSC mobilization with G-CSF and Plerixafor, apheresis, and HSCs/HPCs collection
CD34^+^ HSC/HPC purification and genetic manipulation (lentiviral transfection or gene editor electroporation)
Patient hospitalization and myeloablative conditioning
Infusion of engineered HSCs/HPCs; post-transplantation hospitalization and supportive care
Clinical support, monitoring, and follow-up (including post-transplantation support, evaluation of hematological outcomes of gene-modified hematopoiesis, and evaluation of late effects)

**Table 2:** Main genetic components of lentiviral vectors used for gene therapy of β-hemoglobinopathies.

Main genetic components of lentiviral vectors used for gene therapy of hemoglobinopathies
(1) Therapeutic genes WT-β-globin gene (Vebeglogene Autotemcel) Modified β-globin, β^T87Q^ (Lovotibeglogene) Modified β-globin β^AS3^ (β chain mutant incorporating 3 aminoacidic changes T87Q, E22A and G16D) (Lenti/G-βAS3-FB, GLOBE-AS3) Modified γ-globin, γ^G16D^ (Aru-1801)
(2) Regulatory sequences driving the transgene expression and ensuring high expression in erythroid cells (LCR, Intron 2 β-globin)
(3) Mechanisms to improve safety (self-inactivating viral long-term repeat)
(4) Insulators

**Table 3:** Main gene therapy studies in beta-hemoglobinopathies.

Therapy and Clinical Trial-Gov. No.	Disease	Gene Therapy Approach	Status and Number of Patients
Lov-Cel (Blue Bird Bio) NCT 02140554 HGB-205 and HGB-206	Sickle Cell Disease	Lentiviral globin gene addition (modified β-globin β^T87Q^)	Approved (FDA 2023) 55 patients
Beti-Cel (Blue Bird Bio) NCT 01745120 HGB-204, HGB-205, HGB-207, HGB-212	β°/β°, β^+^/β° Thalassemia	Lentiviral globin gene addition (modified β-globin β^T87Q^)	Approved (EMA 2019, FDA 2022) 40 patients
BCH-BB694 (Boston Children’s Hospital) NCT 03282656	Sickle Cell Disease	Lentiviral BCL11A-shmiR addition	Trial in progress 10 patients
LVV GbG^M^ NCT 02186418	Sickle Cell Disease	Lentiviral globin gene addition (modified γ-globin γ^G16D^)	Trial in progress 7 patients
Exa-Cel (CRISPR Therapeutics/Vertex Pharmaceuticals) NCT 03745287 CLIMB SCD-121/131	Sickle Cell Disease	CRISPR-Cas9 editing of BCL11A enhancer	Approved (EMA 2023, FDA 2023/2024) 44 patients
Exa-Cel (CRISPR Therapeutics/Vertex Pharmaceuticals) NCT 03655678 CLIMB THAL-111	β°/β°, β^+^/β° Thalassemia	CRISPR-Cas9 editing of BCL11A enhancer	Approved 52 patients
Reni-Cel (Editas Medicine) NCT 05444894 EdiThal	β°/β°, β^+^/β°^like^ Thalassemia	Cas 12 editing of the BCL11A binding sited in the γ-globin promoter	Trial in progress 7 patients
Reni-Cel (Editas Medicine) NCT 04853576 Ruby	Sickle Cell Disease	Cas 12 editing of the BCL11A binding sited in the γ-globin promoter	Trial in progress 21 patients
BEAM-101 (Beam Therapeutics) NCT 05456880	Sickle Cell Disease	Base editing of the BCL11A binding site in the γ-globin promoter	Trial in progress 5 patients
BRL-101 (BRL Medicine Inc) NCT 04211480 NCT 04205435	β°/β°, β^+^/β°, β^+^/β^+^ Thalassemia	CRISPR Cas 9 editing of the BCL11A enhancer	Trial in progress (15 patients)

**Table 4:** Different CRISPR-based genome editing strategies used for gene therapy studies of β-hemoglobinopathies.

Gene Editing Strategy Editing Efficacy	Specifity	Main Advantage	Main Limitations	Translational Applications
CRISPR/Cas9 High	Moderate	Cost effectiveness, speed, efficiency, ease of use, flexibility and versatility, multiplexing	Off-target effects, double-strand breaks induced genotoxicity Active only in proliferating cells (HDR)	Disruption of regulators elements regulating HbF synthesis (NHEJ) Correction of point mutations (HDR) Approved for clinical use.
CRISPR/Cas12 High	High	Efficiency in HDR, multiplexing, fewer off-target effects, and more precision in gene targeting than CRISPR/Cas9	Double-strand breaks induced genotoxicity, possible off-target effects	Disruption of regulatory elements regulating HbF synthesis Clinical trials in progress
Base editing Very high	High	Efficient editing No donor template needed, versatile applications, no double-strand breaks, precise correction of single-base changes	Bystander effects, PAM dependency, limited conversion for some base pairs	Base-editing of regulatory elements in HBG1 and HBG2 gene promoters Clinical trials in progress
Prime editing Moderate	Very high	High versatility (base substitutions, small insertions and deletions), precision, reduced off-target effects	Low efficiency (particularly for some DNA sequences), some possible off-target effects Difficult delivery for in vivo studies	Pre-clinical studies
CRISPR a/i	Very high	Efficient activation or inhibition of specific genes	Effect on gene expression insufficient for therapeutic purposes	Pre-clinical studies

## Data Availability

No new data were created or analyzed in this study. Data sharing is not applicable to this article.

## References

[1] Orkin S.H. (2025). The fetal-to-adult hemoglobin switch—Mechanisms and therapy. N. Engl. J. Med..

[2] Huang P., Pesiak C.A., Ren R., Khandros E., Qin K., Keller C.A., Giardine B., Bell H.W., Lan X., Sharma M. (2022). HIC2 controls developmental hemoglobin switching by repressing BCL11A transcription. Nat. Genet..

[3] Bauer D.E., Kamran S.C., Lessard S., Xu J., Fujiwara Y., Lin C., Shao Z., Canver M.C., Smith E.C., Pinello L. (2013). An erythroid enhancer of BCL11A subject to genetic variation determines fetal hemoglobin level. Science.

[4] Masuda T., Wang X., Maedfa M., Canver M.C., Sher F., Funnel A., Fisher C., Suclu M., Martyn G., Norton L. (2016). Transcription factors LRF and BCL11A independently repress expression of fetal hemoglobin. Science.

[5] Piel F., Steinberg M., Rees D.C. (2017). Sickle cell disease. N. Engl. J. Med..

[6] Taher A., Musallam K., Cappellini D. (2021). β-thalassemias. N. Engl. J. Med..

[7] Gluckman E., Cappelli B., Bernaudin F., Labopin M., Volt F., Carreras J., Pinto Simoes B., Ferster A., Dupont S., dela Fuente J. (2017). Sickle cell disease: An international survey of results of HLA-identical sibling hematopoietic stem cell transplantation. Blood.

[8] Kassim A.A., de la Fuente J., Nur E., Wilkerson K.L., Alahmari A.D., Seber A., Bonfim C., Pinto Simoes B., Alzahrani M., Eckrich M.J. (2024). An international learning collaborative phase 2 trial for haploidentical bone marrow transplant in sickle cell disease. Blood.

[9] Kassim A.A., Walters M.C., Eapen M., Smith M., Logan B.R., Solh M., McKinney C., Ross M., Kent M., Abusin G.A. (2025). Haploidentical bone marrow transplantation for sickle cell disease. N. Engl. J. Med. Evid..

[10] Baronciani D., Angelucci E., Potschger U., Gaziev J., Yesilipek A., Zecca M., Orofino M.G., Giardini C., Al-Ahmari A., Marktel S. (2016). Hemopoietic stem cell transplantation in thalassemia: A report from the European Society for Blood and Bone Marrow Transplantation Hemoglobinopathy Registry, 2000–2010. Bone Marrow Transpl..

[11] Xiao H., Huang Q., Lai Y., Liu R. (2025). Haploidentical hematopoietic stem cell transplantation in pediatric transfusion-dependent thalassemia: A systematic review and meta-analysis. Traspl. Cell. Ther..

[12] Jones-Wonni B. (2024). A review of gene therapies for hemoglobinopathies. Hemoglobin.

[13] Butt H., Sathish S., London E., Johnson T.L., Essaewi K., Leonard A., Tisdale J.F., Demirici S. (2025). Genome editing strategies for targeted correction of β-globin mutation in sickle cell disease: From bench to bedside. Mol. Ther..

[14] Fitzhugh C.D., Cordes S., Taylor T., Cotes W., Roskom K., Link M., Hsieh M.M., Tisdale J.F. (2017). At least 20% donor myeloid chimerism is necessary to reverse the sickle phenotype after allogeneic HSCT. Blood.

[15] Frangoul H., Stuits A., Bruce K., Domm J., Carroll C., Aide S., Duckworth M., Evans M., McManus M. (2025). Best practices in gene therapy for sickle cell disease and transfusion-dependent β-thalassemia. Transpl. Cell. Ther..

[16] Baiamonte E., Barone R., Di Stefano R., Lo Iacono M., Spina B., Contino F., Di Maggio R., Sacco M., Vitano A., Feo S. (2015). Hematopoietic stem cell mobilization for gene therapy: The combination of G-CSF+Plerixafor in patients with beta-thalassemia major provides high yields of CD34+ cells with primitive signature. Blood.

[17] Leonard A., Weiss M.J. (2024). Hematopoietic stem cell collection for sickle cell disease gene therapy. Curr. Opin. Hematol..

[18] Oved J.H., Russell A., DeZern A., Prockop S.E., Bonfim C., Sharma A., Purtill D., Lakkaraja M., Biddoli A., Bhoopalan S.V. (2025). The role of conditioning regimen for autologous and ex vivo genetically modified hematopoietic stem cell-based therapies: Recommendations from the ISCT stem cell engineering committee. Cytotherapy.

[19] Poletti V., Mavilio F. (2021). Designing lentiviral vectors for gene therapy of genetic diseases. Viruses.

[20] Ballantine J., Tisdale J.F. (2025). Gene therapy for sickle cell disease: Recent advances, clinical trials and future directions. Cytotherapy.

[21] Sadelain M., Wang C.H., Antoniou M., Grosveld F., Mulligan R.C. (1995). Generation of a high-titer retroviral vector capable of expressing high levels of the human beta-globin gene. Proc. Natl. Acad. Sci. USA.

[22] Miller A.D., Bender M.A., Harris E., Kalako M., Gelinas R.E. (1988). Design of retrovirus vectors for transfer and expression of the human β-globin gene. J. Virol..

[23] Psatha N., Sava P., Georgopoulos G., Psehaudi K., Iwata M., Bloom J., Ulyanova T., Wang H., Kiztsau A., Vasiloudis N.I. (2025). Large-scale discovery of potent, compact and erythroid specific enhancers for gene therapy vectors. Nat. Commun..

[24] Pawliuk R., Westerman K.A., Fabry M.E., Payen E., Tighe R., Bouhassira E.E., Acharya S.A., Ellis J., London I.M., Eaves C.J. (2001). Correction of sickle cell disease in transgenic mouse models by gene therapy. Science.

[25] McCune S.L., Reilly M.P., Chomo M.J., Asakura T., Townes T.M. (1994). Recombinant human hemoglobins designed for gene therapy of sickle cell disease. Proc. Natl. Acad. Sci. USA.

[26] Grimley M., Asnani M., Shrestha A., Felker S., Lutzko C., Arumugam P.I., Witting S., Knight-Madden J., Niss O., Quinn C.T. (2021). Safety and efficacy of Aru-1801 in patients with sickle cell disease: Early results from the phase 1-2 momentum study of a modified gamma globin gene therapy and reduced intensity conditioning. Blood.

[27] Cabriolu A., Odek A., Zamparo L., Yuan H., Leslie C.D., Sadelain M. (2022). Globin vector regulatory elements are active in early hematopoietic progenitor cells. Mol. Ther..

[28] Marshall E. (2011). Gene gemisch cures sickle cell in mice. Science.

[29] Demirci S., Gudmundsdottir B., Li Q., Haro-Mora J.J., Nassehi T., Drysdale C., Yapundich M., Gamer J., Seifuddin F., Tisdale J.F. (2020). βT87Q-globin gene therapy reduces sickle hemoglobin production, allowing for ex vivo anti-sickling activity in human erythroid cells. Methods Clin. Dev..

[30] Ribeil J.A., Hacein-Bey-Abina S., Payen E., Magnani A., Semeraro M., Magrin E., Caccavelli L., Neven B., Bourget P., El Nemer W. (2017). Gene therapy in a patient with sickle cell disease. N. Engl. J. Med..

[31] Magrin E., Semeraro M., Hebert N., Joseph L., Magnani A., Chalumeau A., Gabrion A., Roudaut C., Marouene J., Lfrere F. (2022). Long-term outcomes of lentiviral therapy for the β-hemoglobinopathies: The HGB-205 trial. Nat. Med..

[32] Kanter J., Thompson A.A., Piercey F.J., Hsieh M., Uchida N., Leboulch P., Schmidt M., Bonner M., Guo R., Miller A. (2023). Loco-cel gene therapy for sickle cell disease: Treatment process evolution and outcomes in the initial groups of the HGB-206 study. Am. J. Hematol..

[33] Kanter J., Walters M.C., Krishnamurti L., Mapara M.Y., Kwiatkowski J.L., Rifkin-Zenenberg S., Aygun B., Kasow K.A., Pierciey F.J., Bonner M. (2022). Biologic and clinical efficacy of LentiGlobin for sickle cell disease. N. Engl. J. Med..

[34] Rifkin-Zenenberg S., Kanter J., Kinney M.A., Kwiatkowski J.L., Nickel R.S., Walters M.C., Parikh S., Thompson A., George A.P., Mapara M. (2024). An update on Lovotibeglogene autotemcel (Lovo-cel) for sickle cell disease (SCD) and analysis of early predictors of response to lovo-cel. Blood.

[35] Kanter J., Chawla A., Thompson A.A., Kwiatkowski J.L., Parikh S., Mapara M.Y., Rifkin-Zenenberg S., Aygun B., Kasow K.A., Gupta A.O. (2024). Lovotibeglogene autotemcel gene therapy for sickle cell disease: 60 months follow-up. J. Sickle Dis..

[36] Kinney M.A., Shestopalov I., Christiansen L., Jiang H., Foos M., Elliot H., Chawla A., Piercey F.J. (2024). Predictors of biologic efficacy with Lovotibeglogene autotemcel (Lovo-cel) gene therapy in patients with sickle cell disease. Transpl. Cell. Ther..

[37] Heering W.L., Gallagher M.E., Shah N., Morse K.C., Mladsi D., Dong O.M., Chawrla A., Leiding J., Zhang L., Paramore C. (2024). Cost-effectiveness for Lovotibeglogene autotemcel (Lovo-Cel) gene therapy for patients with sickle cell disease and recurrent vaso-occlusive events in the United States. PharmacoEconomics.

[38] Urbinati F., Fernandez B.C., Masiuk K.E., Poletti V., Hollis R.P., Koziol C., Kaufman M.L., Brown D., Mavilio F., Kohn D.B. (2018). Gene therapy for sickle cell disease: A lentiviral vector comparison study. Human Gene Ther..

[39] Prueksapraopong C., Fernandes A., Fernandez B.C., Roy S., Habtemariam B., Romero Z., Moore T.M., Schiller G.J., Kohn D.B. (2025). Clinical outcomes of Lenti/G-βAS3-FB lentiviral vector gene therapy for sickle cell disease. Transpl. Cell. Ther..

[40] Sobrino S., Joseph L., Magrin E., Chalumeau A., Hebert N., Corsia A., Denis A., Roudaut C., Aussel C., Leblanc O. (2025). Severe inflammation and lineage skewing are associated with poor engraftment of engineered hematopoietic stem cells in patients with sickle cell disease. Nat. Commun..

[41] Perumbeti A., Higashimoto T., Urbinati F., Franco R., Meiselman H.J., Witte D., Lalik P. (2009). A novel human gamma-globin gene vector for genetic correction of sickle cell anemia in a humanized sickle mouse model: Critical determinants for successful correction. Blood.

[42] Grimley M., Davies S.M., Shrestha A., Shova A., Asnani M., Kent M., Sayani F., Quin C., Niss O., Lutzko C. (2025). Lentiviral gene therapy with reduced-intensity conditioning for sickle cell disease: A phase 1-2 trial. Nat. Med..

[43] Thompson A.A., Walters M.C., Kwiatkowski J., Rasko J., Ribeil J.A., Hongeng S., Magrin E., Schiller G.J., Payen E., Semeraro M. (2018). Gene therapy in patients with transfusion-dependent β-thalassemia. N. Engl. J. Med..

[44] Locatelli F., Thompson A.A., Kwiatkowski J., Porter J.B., Thrasher A.J., Hongerng S., Sauer M.G., Thuret I., Lal A., Algeri M. (2022). Betibeglogene autotemcel gene therapy for non-β°/β° genotype β-thalassemia. N. Engl. J. Med..

[45] Kwiatkowski J., Walters M.C., Hongeng S., Yannaki E., Kulozik A.E., Kunz J.B., Sauer M.G., Tharasher A.J., Thuret I., Lal A. (2024). Betibeglogene autotemcel gene therapy in patients with thalassemia-dependent, severe genotype β-thalassemia (HGB-212): A non-randomised, multicentre, single-arm, open-label, single-dose, phase 3 trial. Lancet.

[46] Gibson N.M., Friedman D.F., Elgarten C.W., Haimed A., Khandros E., Worster E., Bardahl J., Wray L., Wang Y., Thompson A.A. (2025). Post-approval, real-world experience with Betibeglogene Autotemcel for transfusion-dependent beta thalassemia. Transpl. Cell. Ther..

[47] Mizza A., Ritsert M.L., Tao G., Thakar H., Labitz S., Heine S., Kosher L., Durken M., Schmitt A., Pavel P. (2025). Gene therapy in transfusion-dependent non-β°/β° genotype β-thalassemia: First real-world experience of beti-cel. Blood Adv..

[48] Marktel S., Scaramuzza S., Cicalese M.P., Giglio F., Galimberti S., Lidonnici M.R., Calbi V., Assanelli A., Bernardo M.E., Rossi C. (2019). Intrabone hematopoietic stem cell gene therapy for adult and pediatric patients affected by transfusion-dependent β-thalassemia. Nat. Med..

[49] Li S., Ling S., Wang D., Wang X., Hao F., Yin L., Yuan Z., Liu L., Zhang L., Li Y. (2024). Modified lentiviral globin gene therapy for pediatric β°/β° transfusion-dependent β-thalassemia: A single-center, single-arm pilot trial. Cell Stem Cell.

[50] Dai X., Li Z., Chen S., Huang Y., Ling S., Wang Q., Wang Q. (2025). Preclinical efficacy and safety evaluation of BD211 autologous CD34^+^ hematopoietic stem cell injection for transfusion-dependent β-thalassemia in NCG-X mice. Front. Cell Dev. Biol..

[51] Brendel C., Negre O., Rothe M., Guda S., Parson G., Harris C., McGuiness M., Abriss D., Tsytsykova A., Klatt D. (2020). Preclinical evaluation of a novel lentiviral vector driving lineage-specific BCL11A knockdown for sickle cell gene therapy. Mol. Ther. Methods Clin. Dev..

[52] Esrick E.B., Lehmann L.E., Biffi A., Achebe M., Brendel C., Ciuculescu M.F., Daley H., MacKinnon B., Morris E., Federico A. (2021). Post-transcriptional genetic silencing of BCL11A to treat sickle cell disease. N. Engl. J. Med..

[53] Esrick E.B., Federico A., Abriss D., Armant M., Boardman K., Brendel C., Ciuculescu M.F., Daley H., Dansereau C., Fernandes A. (2022). Induction of fetal hemoglobin and reduction of clinical manifestations in patients with severe Ahmir-base lentiviral gene therapy for post-transcriptional gene editing of BCL11A: Updated results from pilot and feasibility trial. Blood.

[54] Esrick E.B., Lehmann L.E., Federico A., Daley H., Dansereau C., DeOliveira S., Everett J., Kao P.C., Liu B., Moore T. (2025). Long-term follow-up of the first in human post-transcriptional genetic silencing of BCL11A in sickle cell disease in a phase 1 pilot and feasibility study. Blood.

[55] Jimenez-Kurlander L., Kao P.C., Morris E., Federico A., Moore T., DeOliveira S., Fernandes A., Kohn D.B., London W.B., Williams D.A. (2023). Patient and parent-reported outcomes post-treatment with Shmir-based lentiviral gene therapy for sickle cell disease. Blood.

[56] De Souza D.C., Hebert N., Esrick E.B., Ciuculescu M.F., Archer N.M., Armant M., Audureau É., Brendel C., Di Caprio G., Galactéros F. (2023). Genetic reversal of the globin switch concurrently modulates both fetal and sickle hemoglobin and reduces red cell sickling. Nat. Commun..

[57] Spencer Chapman M.S., Cull A.H., Ciuculescu M.F., Esrick E.B., Mitchell E., Jung H., O’Neill L., Roberts K., Fabre M.A., Williams N. (2023). Clonal selection of hematopoietic stem cells after gene therapy for sickle cell disease. Nature.

[58] Xue C., Greene E.C. (2021). DNA repair pathway choices in CRISPR-Cas9-mediated genome editing. Trends Genet..

[59] Rees H.A., Liu D.R. (2018). Base editing: Precision chemistry on the genome and transcriptome of living cells. Nat. Rev. Genet..

[60] Kostamo Z., Ortega M.A., Xu C., Feliciano P.R., Budak E., Lam D., Winton V., Jenkins R., Venugopal A., Zhang M. (2025). Base editing HbS to HbG-Makassar improves hemoglobin function supporting its use in sickle cell disease. Nat. Commun..

[61] Radtke S., Fields E., Swing K., Kanestrom G., Yen J.S., Pande D., Enstrom M.R., Humbert O., Weiss M.J., Liu D.R. (2025). Engraftment and persistence of HBB base-edited hematopoietic stem cells in nonhuman primates. Sci. Transl. Med..

[62] Mayuranathan T., Newby G.A., Feno R., Yao Y., Mayberry K.D., Lazzarotto C.R., LI Y., Levine R.M., Nimmagadda N., Dempsey E. (2023). Potent and uniform fetal hemoglobin induction via base editing. Nat. Genet..

[63] Han W., Qiu H.Y., Sun S., Fu Z.C., Wang Q.Q., Qian X., Wang L., Zhai X., Wei J., Wang Y. (2023). Base editing of the HBG promoter induces potent fetal hemoglobin expression with no detectable off-target mutations in human HSCs. Cell Stem Cell.

[64] Fontana L., Martinucci P., Amistadi S., Felix T., Mombled M., Tachtsidi A., Corre G., Chalumeau A., Hardouin G., Martin J. (2025). Multiplex base editing of BCL11A regulatory elements to treat sickle cell disease. Cell Rep. Med..

[65] Rajendiran V., Devaraju N., Haddad M., Ravi N.S., Panigrahi L., Paul J., Gopalakrishnan C., Wyman S., Ariudainambi K., Mahalingam G. (2024). Base editing of key residues in the BCL11A-XL-specific zinc finger domains derepresses fetal globin expression. Mol. Ther..

[66] Chen P.J., Liu D.R. (2023). Prime editing for precise and highly versatile genome manipulation. Nat. Rev. Genet..

[67] Chauhan V., Sharp P.A., Langer R. (2025). Engineered prime editors with minimal genomic errors. Nature.

[68] Everette K.A., Newby G.A., Levine R.M., Mayberry K., Jang Y., Mayuranathan T., Nimmagadda N., Dempsey E., Li Y., Bhoopala N. (2023). Ex vivo prime editing of patient haematopoietic stem cells rescues sickle-cell disease phenotypes after engraftment in mice. Nat. Biomed. Eng..

[69] Chalumeau A., Dames M.B., Fontana L., Amistadi S., Anatoniou P., Loganathan P., Mombled M., Corre G., Peterka M., Amendola M. (2025). A prime editing strategy to rewrite the γ-globin promoters and reactivate fetal hemoglobin for sickle cell disease. Blood.

[70] Fiumara M., Ferrari S., Omer-Javed A., Beretta S., Albano L., Canarutto D., Varesi A., Gaddoni C., Brombin C., Cuganta F. (2024). Genotoxic effects of base and prime editing in human haematopoietic stem cells. Nat. Biotechnol..

[71] Hwang G.H., Lee S.H., Oh M., Kim S., Habib O., Jang H.K., Kim H.S., Kim Y., Kim C.H., Kim S. (2025). Large DNA deletions occur during DNA repair at 20-fold lower frequency for base editors and prime editors than for Cas9 nucleases. Nat. Biomed. Eng..

[72] Gilbert L.A., Larson M.H., Morsut L., Liu Z., Brar G.A., Torres S.E., Stern-Ginossar N., Brandman O., Whitehead E.H., Doudna J.A. (2013). CRISPR-mediated modular RNA-guided regulation of transcription in eukaryotes. Cell.

[73] Dominguez A.A., Lim W.A., Qi L.S. (2016). Beyond editing: Repurposing CRISPR-Cas9 for precision genome regulation and interrogation. Nat. Rev. Mol. Cell Biol..

[74] Qi L.S., Larson M.H., Gilbert L.A., Doudna J.A., Weissman J.S., Arkin A.P., Lim W.A. (2021). Repurposing CRISPR as an RNA-guided platform for sequence-specific control of gene expression. Cell.

[75] Bell H.W., Feng R., Shah M., Yao Y., Douglas J., Doerfler P.A., Mayuranathan T., O’Dea M.F., Li Y., Wang Y.D. (2025). Removal of promoter CpG methylation by epigenome editing reverses HBG silencing. Nat. Commun..

[76] Ye L., Wang J., Tan Y., Beyer A.I., Zie F., Muench M.O., Kan Y.W. (2016). Genome editing using CRISPR-Cas9 to create the HPFH genotype in HSPCs: An approach for treating sickle cell disease and β-thalassemia. Proc. Natl. Acad. Sci. USA.

[77] Wu Y., Zeng J., Roscoe B.P., Liu P., Yao Q., Lazzarotto C., Clement K., Cole M.A., Luk K., Baricordi C. (2019). Highly efficient therapeutic gene editing of human hematopoietic stem cells. Nat. Med..

[78] Wang K., Wang J., Feng R., Dudnyk K., Kim Y.J., Lim J.Y., Lee M., Zhang Y., Gao X., Cheng Y. (2025). Silencing of BCL11A by disrupting enhancer-dependent epigenetic insulation. Blood.

[79] Frangoul H., Althshuler D., Cappellini M.D., Chen Y.S., Domm J., Eustace B.K., Foeli J., de la Fuente J., Grupp S., Handgretinger R. (2021). CRISPR-Cas9 gene editing for sickle cell disease and β-thalassemia. N. Engl. J. Med..

[80] Locatelli F., Lang P., Wall D., Meisel R., Carbacioglu S., Li A.M., de la Fuente J., Shah A.J., Carpentier B., Kwiatkowski J.L. (2024). Examglogene autotemcel for transfusion-dependent β-thalassemia. N. Engl. J. Med..

[81] Frangoul H., Locatelli F., Lang P., Meisel R., Wall D.A., Carbacioglu S., Li A., de la Fuente J., Shah A.J., Carpenter B. (2023). Durable clinical benefits in transfusion-dependent β-thalassemia with Examglogene Autotemcel. Transplant. Cell. Ther..

[82] Locatelli F., Meisel R., Carbacioglu S., de la Fuente J., Algeri M., Ruppechjt J., Kuo K., Shah A., Lang P., Merkeley H. (2025). Correction of ineffective erythropoiesis and durable clinical benefit with exagamglogene autotemcel for transfusion-dependent β-thalassemia. Blood.

[83] Fu B., Liao J., Chen S., Li W., Wang Q., Hu J., Yang F., Hsiao S., Jiang Y., Wang L. (2022). CRISPR-Cas9-mediated gene editing of the BCL11A enhancer for pediatric β°/β° transfusion-dependent β-thalassemia. Nat. Med..

[84] Zheng B., Liu R., Zhang X., Fu B., Xu Y., Shi J., Feng X., Wang L., Wang C., Liang R. (2023). Efficacy and safety of BRL-101, CRISPR-Cas9-mediated gene editing of the Bcl11A enhancer in transfusion-dependent β-thalassemia. Blood.

[85] Zheng B., Liu R., Zhang X., Fu B., Xu Y., Shi J., Feng X., Li D., Wu Y., Liu M. (2024). Efficacy and Safety of BRL-101, CRISPR-Cas9-Mediated Gene Editing of the Bcl11A Enhancer in Transfusion-Dependent Beta-Thalassemia. EHA Library.

[86] Frangoul H., Locatelli F., Sharma A., Bhatia M., Mapara M., Liem R.I., Telfer P., Shah A.J., Cavazzana M., Corbacioglu S. (2024). Examglogene autotemcel for severe sickle cell disease. N. Engl. J. Med..

[87] Grupp S.A., Locatelli F., Sharma A., Bhatia M., Mapara M., Liem R.I., Wall D.A., Molinari L., Dedeken L., Kuo K. (2025). Durable clinical benefits in severe sickle cell disease with exagamglogene autotemcel. Transl. Cell. Ther..

[88] Grupp S., Locatelli F., Sharma A., Bhatia M., Mapara M., Liem R., Dedeken L., Molinari L., Eckrich M., Kuo K. (2025). Long-term follow-up demonstrates durable clinical benefits of examglogene autotemcel for sickle cell disease with recurrent vaso-occlusive crises: Final results of Climb SCD-121. Blood.

[89] Sharma S.A., Locatelli F., Bhatia M., Molinari L., Mapara M., Liem R., Wall D.A., Molinari L., Dedekan L., Wall D. (2025). Improvements in health-related quality of life in patients with severe sickle cell disease after examglogene autotemcel. Blood Adv..

[90] Frangoul H., de la Fuente J., Algeri M., Chopra Y., Amrolia P., Sharma A., Meisel R., Cappellini M.D., Carbacioglu S., Kattamis A. (2025). First results of examglogene autotemcel in pediatric patients aged 5-11 years with transfusion-dependent β-thalassemia or sickle cell disease with recurrent severe vaso-occlusive crises. Blood.

[91] Ye D., Chen M., Zhu Y., Feng X., Xu L., Huang H. (2025). Comprehensive regulation of γ-globin expression by epigenetic modification s and protein post-translational modifications. Clin. Epigenetics.

[92] Wongabirisuth C., Innachai P., Saisawang C., Tabsuwan A., Jearawirslyaparisan N., Kaewprommal P., Piryapoagsa J., Chiangyong W., Anurathapani U., Sandeis D. (2025). Disrupting ZBTB7A or BCL11A binding sites reactivates fetal hemoglobin in erythroblasts from healthy and β°/HbE individuals. Sci. Rep..

[93] Traxler E.A., Yao Y., Wang Y.D., Woodard K.J., Kurita R., Nakamura Y., Hughes J.R., Hardison R.C., Blobel G.A., Li C. (2016). A genome editing strategy to treat β-hemoglobinopathies that recapitulates a mutation associated with a benign genetic condition. Nat. Med..

[94] Métais J.Y., Doerfler P.A., Mayuranathan T., Bauer D.E., Fowler S.C., Hsieh M.M., Katta V., Kerwala S., Lazzarotto C.R., Luk K. (2019). Genome editing of HBG1 and HBG2 to induce fetal hemoglobin. Blood Adv..

[95] Sharma A., Boelens J.J., Cancio M., Hanking J.S., Bhad P., Azizy M., Lewandoski A., Zhao X., Chitnis S., Peddinti R. (2023). CRISPR-Cas9 editing of the HBBG1 and HBBG2 promoters to treat sickle cell disease. N. Engl. J. Med..

[96] Gupta A.O., Sharma A., Frangoul H., Dalai J., Kanter J., Alavi A., DiPersio J., Eapen M., Jaroscak J.J., Ayala E. (2024). Initial results from the BEACON clinical study: A phase 1-2 study evaluating the safety and efficacy of a single dose of autologous CD34^+^ base edited hematopoietic stem cells (BEAM-101) in patients with sickle cell disease with severe vaso-occlusive crises. Blood.

[97] Chockalingram P.S., Chen G., Minella A.C., Chen Y., Shehan V., Zhang N., Armant M., Zaidi A.U., Goodrich R., Hines P.C. (2024). Impact of BEAM-101 treatemnt on red blood cell hemoglobin expression, rheology and sickling properties: Initial data from the BEACON phase 1-2 study of autologous CD34^+^ base edited hematopoietic stem cells in sickle cell disease. Blood.

[98] Gupta A.O., Sharma A., Frangoul H., Dalai J., Kanter J., Alavi A., DiPersio J., Eapen M., Jaroscak J.J., Ayala E. Base editing for sickle cell disease: Ongoing results from the Beacon study evaluating the safety and efficacy of BEAM-101, the first base-edited autologous CD34^+^ HSPC one-time cell therapy. Proceedings of the Abstracts of the 30th Annual Congress of the European Hematology Association (EHA 2025).

[99] Gupta A., Sharma A., Frangoul H., Kanter J., Mapara M., Dalai J., Alavi A., Jaroscak J., Ayala E., DiPersio J. (2025). Robust HbF induction and improvement of anemia and hemolysis with base editing in sickle cell disease: Safety and efficacy findings from the ongoing BEACON study. Blood.

[100] Wang L., Xue W., Zhang H., Gao R., Qiu H., Wei J., Zhou L., Lei Y.N., Wu X., Li X. (2021). Eliminating base-editor-induced genome-wide and transcriptome-wide off-target mutations. Nat. Cell Biol..

[101] Wang L., Li P., Wang Y., Lan K., Zheng H., Zhu D., Zhang Y., Guo R., Ma H., He J. (2023). Development of best-in-class gene editing therapy for β-hemoglobinopathies using innovative transformer base editor (tBE). Blood.

[102] Lai Y., Liu R., Wang L., Ma X.K., Li Y., Yang G., Shi L., Wei J., Wei Z., Zhou X. (2024). Rapid, efficient and durable fetal hemoglobin production following CS-101 treatment in transfusion-dependent β-thalassemia participants: An autologous, ex vivo edited CD34+ stem cell product using the innovative transformer base editor (tBE). Blood.

[103] Lai Y., Liu R., Wang L., Ma X.K., Li Y., Yang G., Shi L., Wei J., Wei Z., Zhou X. Treatment of patients with severe transfusion-dependent β-thalassemia with CS-101, an autologous, ex vivo edited, CD34+ hematopoietic stem cell product using innovative transformer base editor (TBE). Proceedings of the Abstracts of the 30th Annual Congress of the European Hematology Association (EHA 2025).

[104] Chen J., Lai Y., Zhai X., Zhao W.L., Liu R., Qian X., Tang W., Wang L., Ma X.K., LI Y. (2025). Rapid, efficient and durable fetal hemoglobin production following CS-101 treatment in transfusion-dependent β-thalassemia participants; an autologous, ex vivo edited CD34^+^ stem cell product using the innovative transformer base editor (tBE). Blood.

[105] Paul B., Montoya G. (2020). CRISPR-Cas12a: Functional overview and applications. Biochem. J..

[106] De Dreuzy E., Haeth G., Zuris J.A., Sousa P., Viswanathan R., Scott S., Da Silva J., Ta T., Copehart S., Wang T. (2019). EDIT-301: An experimental autologous cell therapy comprising Cas12a-RNP modified mPB-CD34+ cells for the potential treatment of SCD. Blood.

[107] Hanna R., Frangoul H., McKinney C., Pineiro L., Mapara M., Dalal J., Rangarajan H., Atkins H., Chang K.H., Mei B. (2023). As Cas12a gene editing of HBG1/2 promoters with EDIT-301 results in rapid and sustained normalization of hemoglobin with increased fetal hemoglobin in patients with severe sickle cell disease and transfusion-dependent beta-thalassemia. Blood.

[108] Hanna R., Frangoul H., Pineiro L., McKinney C., Mapara M., Dalai J., Rangarajan H., Atkins H., Bhatia M., Chellapandian D. (2025). CRISPR-Cas12a gene editing of the HBG 1-2 promoters leads to sustained normalization of total hemoglobin and increased fetal hemoglobin in patients with severe sickle cell disease: Updated results from the RUBY study. Blood.

[109] Frangoul H., Hanna R., Walters M.C., Chang K.H., Jaskolka M., Kim K., Mei B., Afonja O., Thompson A. (2024). Reni-Cel, an investigational As Cas12a gene-edited cell medicine, led to substantial engraftment, increased hemoglobin, and reduced transfusion dependence in patients with transfusion-dependent beta-thalassemia treated in the Edithal trial. Blood.

[110] Uchida N., Li L., Nassehi T., Drysdale C.M., Yapundich M., Gasner J., Haro-Mora J., Demirci S., Leonard A., Bonifacino A.C. (2021). Preclinical evaluation for engraftment of CD34^+^ cells gene-edited at the sickle disease locus in xenograft mouse and non-human primate. Cell Rep. Med..

[111] Kanter J., DiPersio J.F., Leavey P., Shyr D.C., Thompson A.A., Porteus M.H., Intondi A., Lahiri P., Dever D.P., Petrusich A. (2021). Cedar trial in progress: A first in human, phase 1-2 study of the correction of a single nucleotide mutation in autologous HSCs (GPH 101) to convert HbS to HbA for treating severe SCD. Blood.

[112] Shyr D.C., Lowsky R., Miller W., Schroeder M.A.-, Bucholz T., Dougall K., Intondi A., Charles A., Leher J., Lehrer J. (2023). One year follow-up on the first patient treated with Nula-Cel: An autologous CRISPR/Cas9 gene corrected CD34+ cell product to treat sickle cell disease. Blood.

[113] Lee B., Gin A., Wu C., Singh K., Grice M., Mortlock R., Abraham D., Fan X., Zhou Y., Aljanahi A. (2024). Impact of CRISPR/HDR editing versus lentiviral transduction on long-term engraftment and clonal dynamics of HSPCs in rhesus macaques. Cell Stem Cell.

[114] Lessard S., Rimmelé P., Ling H., Moran K., Vieira B., Lin Y.D., Rajani G.M., Hong V., Reik A., Boismenu R. (2024). Zinc finger nuclease-mediated gene editing in hematopoietic stem cells results in reactivation of fetal hemoglobin in sickle cell disease. Sci. Rep..

[115] Li C., Georgakopoulou A., Newly G.A., Chen P.J., Everette K.A., Paschoudi K., Vlachaki E., Gil S., Anderson A.K., Koob T. (2023). In vivo HSC prime editing rescues sickle cell disease in a mouse model. Blood.

[116] Li C., Anderson A.K., Ruminski P., Kapova D., Kidem H.P., DiPersio J.F., Lieber A. (2024). A simplified G-CSF-free procedure allows for in vivo HSC gene therapy of sickle cell disease in a mouse model. Blood Adv..

[117] Li C., Georgakopoulou A., Paschoudi K., Anderson A.K., Huang L., Gil S., Giannaki M., Vlachaki E., Newby G.A., Liu D.R. (2024). Introducing a hemoglobin G-Makassar variant in HSCs by in vivo base editing treats sickle cell disease in mice. Mol. Ther..

[118] Breda L., Papp T.E., Triebwasser M.P., Yadegari A., Fedorky M.T., Tanaka N., Abdulmalik O., Pavani G., Wang Y., Grupp S.A. (2023). In vivo hematopoietic stem cell modification by mRNA delivery. Science.

[119] Lian X., Chatterjee S., Dilliard S.A., Moore S., Xiao Y., Bian X., Yamada K., Sung Y.C., Levine R.M., Mayberry K. (2024). Bone-marrow-homing lipid nanoparticles for genome editing in diseased and malignant hematopoietic stem cells. Nat. Biotechnol..

[120] Xu S., Liang D., Wang Q., Cheng Y., Xie D., Gui Y., Zhang H., Feng C., Zhao F., Ren W. (2025). In vivo genome editing of human hematopoietic stem cells for treatment of blood disorders using mRNA delivery. Nat. Biomed. Eng..

[121] Liao J., Chen S., Hsiao S., Jiang Y., Yang Y., Zhang Y., Wang X., Lai Y., Bauer D.E., Wu Y. (2023). Therapeutic base adenine editing of human hematopoietic stem cells. Nat. Commun..

[122] Milani M., Fabiano A., Perez-Rodriguez M., Hernandez R.J., Zecchillo A., Zonari E., Ottonello S., Basso-Ricci L., Canepari C., Volpin M. (2025). In vivo hematopoietic stem cell gene therapy enabled by postnatal trafficking. Nature.

[123] Milani M., Annoni A., Moalli F., Liu T., Cesana D., Calabria A., Bartolaccini S., Biffi M., Russo F., Visigalli I. (2019). Phagocytosis-shielded lentiviral vectors improve liver gene therapy in nonhuman primates. Soc. Transl. Med..

[124] Charlesworth C.T., Homma S., Amaya A.K., Dib C., Vaidyanathan S., Tan T.K., Miyauchi M., Nakauchi Y., Suchy F.P., Wang S. (2025). Highly efficient in vivo hematopoietic stem cell transduction using an optimized self-complementary adeno-associated virus. Mol. Ther. Methods Clin. Dev..

[125] Karimzadeh A., Kim R., Garcia V., Fiorea M., Peacker B.L., Kobayashi S., Watkins D., Messemer K., Zheng J., Bauer D.E. (2025). In situ gene editing of hematopoietic stem cells via AAV-delivered CRISPR guide RNAs. Blood Adv..

[126] Bhoorasingh M., Vail E., Yoon Y., Black R., Reddy M., Jeong S. (2025). Engineered extracellular vesicles for in vivo therapy in sickle cell disease. Blood.

[127] Tozzi L., Schiroli G., Heshmati Y., Cao Y., Monte T., Wangweerawong A., Rottman J.B., Palchaudhuri R., Ribeil J.A., Manis J. (2024). In vivo HSC gene editing for correction of the sickle cell mutation using RNA gene writers. Blood.

[128] Heshmati Y., Schiroli G., Tozzi L., Monte M., Beytour N., Wangweerawong A., Rottman J., Salomon W.E., Palchaudhuri R., Wang J. (2025). In vivo correction of the sickle cell disease mutation in hematopoietic stem cells using RNA gene writers. Blood.

[129] Raguram A., Banskota S., Liu D.R. (2022). Therapeutic in vivo delivery of gene editing agents. Cell.

[130] Jones R.J., Kassim A., Brodsky R.A., De Baun M. (2025). Is allogeneic transplantation for sickle cell disease still relevant in the era of gene therapy?. Blood Adv..

[131] Santorone S., Angelini S., Natale A., Vaddinelli D., Spadano R., Casciani P., Papola F., DiLembo E., Iannetti G., Di Bartolomeo P. (2022). Survival and late effects of hematopoietic cell transplantation in patients with thalassemia major. Bone Marrow Transpl..

[132] Santorone S., Pepe A., Meloni A., Natale A., Pistoia L., Olioso P., Papalinetti G., Cuccia L., Spasiano A., Lisi R. (2018). Secondary solid cancer following hematopoietic transplantation in patients with thalassemia major. Bone Marrow Transpl..

[133] De Franceschi L., Locatelli F., Rees D., Chabannon C., Dalle J.H., Rivella S., Iolascon A., Lobitz S., Abboud M.R., de la Fuente J. (2025). Selecting patients with sickle cell disease for gene addition or gene editing-based therapeutic approaches: Report on behalf of a joint EHA specialized working group and EBMT hemoglobinopathies working party consensus conference. HemaSphere.

[134] Sharma A., John T. (2025). To pursue gene therapy or not? Is it feasible after graft failure in allogeneic hematopoietic cell transplant recipients?. Blood Adv..

[135] Kelkar A.H., Achebe M.O., Hantel A. (2025). An ethical allocation scheme for scarce gene therapies in sickle cell disease and transfusion-dependent β-thalassemia. Blood Adv..

[136] John T., Czechowiez A. (2025). Clinical hematopoietic stem cell-based gene therapy. Mol. Ther..

[137] Boulad F., Maggio A., Wang X., Moi P., Acuto S., Kogel F., Takpradit C., Mansilla-Soto J., Cabriolu A., Odak A. (2022). Lentiviral globin gene therapy with reduced-intensity conditioning in adults with β-thalassemia: A phase 1 trial. Nat. Med..

[138] Hsieh M.M., Bonner M., Pierciey F.J., Uchida N., Rottman J., Demopoulos L., Schmidt M., Kanter J., Walters M.C., Thompson A.A. (2020). Myelodysplastic syndrome unrelated to lentiviral vector in patient treated with gene therapy for sickle cell disease. Blood Adv..

[139] Goyal S., Tisdale J., Schmidt M., Kanter J., Jaroscak J., Whitney D., Bitter H., Gregory P.D., Parsons G., Foos M. (2022). Acute myeloid leukemia case after gene therapy for sickle cell disease. N. Engl. J. Med..

[140] Liggett L.A., Cato L.D., Weinstock J.S., Zhang Y., Nourale S.M., Gladwin M.T., Garrett M.E., Ashley-Koch A., Telen M.J., Custer S. (2022). Clonal hematopoiesis in sickle cell disease. J. Clin. Investig..

[141] Pincez T., Lee S., Ilboudo Y., Preuss M., Hung d’Alexandry d’Orengiani A.L., Bartolucci P., Galacteros F., Loly P., Bauer D.E., Loos R. (2021). Clonal hematopoiesis in sickle cell disease. Blood.

[142] Solomou E.E., Delaporta P., Stamatia L., Katsika E.V., Catzieleftheriou M., Toutoudaki K., Glentis S., Stamatopoulos K., Catzidimitriou A., Kattamis A. (2022). High incidence of clonal hematopoiesis in transfusion-dependent thalassemia patients. Blood.

[143] De Luna G., Cretin J., Redjoul R., Beckerich F., Habiobi A., Menouche D., Hebert N., Giovannangeli C., Maury S., Bartolucci P. (2025). Screening for clonal hematopoiesis in patients with β-hemoglobinopathies who are candidates to transplant approaches. Blood.

[144] Weeks L., Fitzhugh C., Pollock S., Osei M., Rickles-Young M., Murdock M., Townsend M., Reilly C., Dinardo C., Sabino E. (2025). Sickle cell disease is associated with early onset clonal hematopoiesis involving DNA damage response pathway mutations. Blood.

[145] Zeng J., Nguyen M.A., Liu P., Ferreira da Silva L., Levesque S., Lin L.Y., Justus D.G., Petri K., Clement K., Porter S.N. (2025). Gene editing without ex vivo culture evades genotoxicity in human hematopoietic cells. Cell Stem Cell.

[146] Demirci S., Zeng J., Pelchaudhuri R., Wu C., Abraham D.M., Hayal T.B., Essawi K., Nguyen M.A., Stasula U., Chu R. (2025). BCL11A +58/+55 enhancer-editing facilitates HSPC engraftment and HbF induction in rhesus macaques conditioned with a CD45 antibody-drug conjugate. Cell Stem Cell.

[147] Felder A.K., Tjalsma S., Verhagen H., Majied R., Verstegen M., Verheul T., van Haren J., Mohnani R., Gremmen R., Krijer P. (2025). Reactivation of developmentally silenced globin genes through forced linear recruitment of remote enhancers. Blood.

[148] Hardouin G., Martinucci P., Scaramuzza S., Antoniou P., Corradi F., Tachtsidi A., Corre G., Mombled M., Chermont J., Manceau S. (2025). Base editing of β°-thalassemia mutations as a therapeutic strategy for severe β-hemoglobinopathies. Sci. Trasl. Med..

[149] Butt H., Sathish S., London E., Le A., Li Q., Gudmundsdottir B., Lee D.Y., Burke E.V., Yates B.P., Liu D.R. (2025). Comparative analysis of CRISPR-Cas9, lentiviral transduction, and base editing for sickle cell disease in a murine model. Blood Adv..

